# A simple satisficing model

**DOI:** 10.1371/journal.pone.0275339

**Published:** 2022-10-10

**Authors:** Erlend Dancke Sandorf, Danny Campbell, Caspar Chorus

**Affiliations:** 1 School of Economics and Business, Norwegian University of Life Sciences, Ås, Norway; 2 Economics Division, Stirling University Management School, Scotland, United Kingdom; 3 Transport and Logistics Group, Faculty of Technology, Policy and Management, Technical University of Delft, Delft, The Netherlands; Texas A&M University, UNITED STATES

## Abstract

Economic theory is built on the assumption that people are omniscient utility maximizers. In reality, this is unlikely to be true and often people lack information about all alternatives that are available to them; either because the information is unavailable or that the cost of searching for and evaluating that information is high. In this paper, we develop a simple and tractable model that captures satisficing behavior. We show that the model can retrieve consistent parameters under a large range of experimental conditions. We test our model on synthetic data and present an empirical application. We discuss the implications of our results for the use of satisficing choice models in explaining choice.

## Introduction

Economic theory is built on the assumption that people are omniscient utility maximizers. That they have complete information about all available options, knowledge of their preferences and the ability to calculate their expected utility from choosing any one option. While these assumptions are useful for welfare analysis, they may not fully describe how people make choices in real life. Indeed, people routinely make decisions that cannot readily be described by the standard model of rationality [1–9]. Simon [1] argued that people lack the memory and cognitive abilities to be perfectly rational; or that they actively choose to avoid the (significant) cognitive effort associated with searching for and evaluating all possible alternatives available to them. Instead, people are “boundedly rational”. The idea of bounded rationality is built on the premise that people rarely have complete information about alternatives nor do they have perfect knowledge of their preferences, but learn about both through the (costly) search for information and deliberation [1, 10, 11].

The search for information about alternatives has interesting implications for choice. First, it is at odds with the standard model of expected utility maximization. That model assumes that people have complete information about all available alternatives. Let us call this collection of alternatives the grand choice set, and it includes every single possible (relevant) alternative. When an individual searches for alternatives, their choice set is growing with one alternative per period of search. Let us call these smaller choice sets, which are proper subsets of the grand choice set, their consideration sets. In optimal search theory, this process of growing the consideration set would continue until the expected gains from continuing to search would be less than or equal to the marginal cost of continuing to search [12, 13]. At this point, a choice will be made among the alternatives present in the consideration set. This choice can still be compensatory and made by an individual maximizing utility. However, it is not necessarily true that the alternative maximizing utility in the consideration set is the ‘global’ utility maximizing alternative, indicating that the chosen alternative may be suboptimal. Furthermore, this process also implies that people can continue to search for additional alternatives even after a satisfactory or utility maximizing alternative has been found. Indeed, Stüttgen et al. [14] find evidence that people keep searching after having found a satisfactory alternative, which the authors argue may be a way of confirming that the choice they made was a good one. To use the terminology in Aribarg et al. [7], this would be a typically sequential and stochastic process.

That said, Simon [1] argues that even this might be too complex a decision for people to make and that decisions might not even involve maximization of some function at all. Rather people make a sequence of decisions based on whether or not the utility of the current alternative exceeds some threshold utility. We formally define the threshold utility in the next section of the paper. We also note that it is not necessary to work within the framework of utility, but as economists, we find it useful to do so. As such, the decision process is one in which each alternative is evaluated sequentially and the first one exceeding the threshold utility is chosen. For example, imagine you are on holiday in a new city and are going out for dinner. How do you decide where to go? Do you consider every restaurant in the city and choose the one that gives the highest expected utility based on the menu, distance from the hotel, renown and service? Probably not. It is more likely that you engage in a type of satisficing process where you evaluate a set of restaurants sequentially, and subject to how hungry you are, choose the first one that has an acceptable menu, distance to the hotel, renown and service. The utility derived from the marginal restaurant meeting all these minimum requirements (later: aspiration levels) is the threshold utility and the chosen restaurant is the first one giving a utility higher than this, i.e., exceeds the minimum on at least one attribute level. We want to make clear that satisficing differs from other non-compensatory decision rules such as lexicographic choices and elimination-by-aspects. Continuing with our restaurant example: A decision maker choosing lexicographically will choose a restaurant based on their perceived most important attribute; whereas a decision maker who eliminates-by-aspects will gradually reduce the number of restaurants by iteratively excluding those that do not meet minimum acceptable attribute cut-off levels until a single chosen restaurant remains. As stated above, a satisficing choice may or may not be utility maximizing. If the first satisfactory restaurant encountered happens to be the one that gives the highest global utility, then that choice is also utility maximizing. However, any other choice is by definition satisfactory, but not maximizing [15, 16].

Several authors have found evidence that people in experimental settings make decisions that are (partly) consistent with a satisficing decision rule [8, 11, 17–20] (for a more thorough review of the literature see the review sections in Sandorf and Campbell [8], González-Valdés and Ortúzar [19], Manski [10], Papi [16] and Aribarg et al. [7]). For example, Caplin et al. [21] develop a theoretical model of search and satisficing, which they test on choice process data that allows the experimenter to track participants’ choices. They find that when people work under a time constraint, they tend to choose the first option that meets their aspiration level. Reutskaja et al. [18], on the other hand, use eye-tracking, as opposed to choice process data, to determine the order in which alternatives were evaluated. They impose a strict time constraint with a monetary penalty for participants who spent more time than they were allotted, and they find that participants’ choices are partly consistent with satisficing. In a different eye-tracking study, Stüttgen et al. [14] find that participants stop searching for new alternatives when they find one that is satisfactory. Manski [10] explores satisficing in the context of deliberation costs. He shows that if the cost of learning about one’s preferences is prohibitively high, then people will resort to a no deliberation strategy, which involves choosing the first alternative they encounter. If costs are positive, but not prohibitive, people will engage in satisficing behavior, and if costs are low, possibly zero, people will optimize, e.g., maximize utility. Satisficing decisions are not limited to the domain of frequent or infrequent decisions. Gigerenzer [22] argues that moral decisions can be so difficult and mentally taxing that satisficing is particularly prevalent in this context.

Herbert Simon’s idea of satisficing has been quite successful but has received limited interest from choice modelers. To the authors’ knowledge, only a few papers have developed models that can identify satisficing behavior in more traditional discrete choice data (e.g., see Sandorf and Campbell [8] and González-Valdés and Ortúzar [19]), and none are necessarily readily implemented within existing software. González-Valdés and Ortúzar [19] describe a “stochastic” satisficing model where an alternative is deemed satisfactory if all attribute levels are deemed satisfactory. Their model dismisses the notion of utility completely and relies on a set of acceptability functions. The model is stochastic in the sense that it assumes a random starting point and direction to model the search path. To overcome the implications of findings by Stüttgen et al. [14] they assume that people choose the first acceptable alternative and do not continue to search. Sandorf and Campbell [8] on the other hand developed a model to systematically explore the use of the satisficing decision rule. Specifically, they considered 944 possible satisficing rules (threshold utilities) and allowed respondents to revise their rules throughout the choice sequence, which is in line with aspiration adaption [23]. While only a minority chose according to a satisficing rule, the implications for welfare estimates were significant.

Any model trying to capture satisficing behavior needs to make assumptions concerning i) when information search starts, ii) the direction of information search, and iii) when search stops. In addition, a properly specified model needs to address potential search costs. The assumptions i)-iii) implies that the search path is observable. This is generally not the case with standard discrete choice data. Eye-tracking can help, but to fully observe the search path, mechanisms to detect and track this must be in place at the design and implementation stages. If the search path is observable, then determining i)-iii) is trivial.

Sandorf and Campbell [8] developed and applied a satisficing model to data obtained from a standard stated preference survey that was not designed to detect satisficing behavior. To estimate the model they had to assume that people evaluated alternatives in a particular order. Specifically, they assumed that alternatives were evaluated in the direction of reading, i.e., from left to right. In this paper, we develop a simple satisficing model that accommodates choosing the first alternative with utility exceeding some threshold level of utility. This threshold utility is estimated along with the marginal utility parameters. To test the performance of our model, we run a series of Monte-Carlo simulations on data generated assuming people are utility maximizing, i.e., a standard multinomial logit model, and data generated assuming people are satisficing, i.e., the simple satisficing model. In addition to satisficing, we also allow for other secondary decision rules, for example, choose the first and choose the last. We show that our model can retrieve the true parameters under various assumptions about the level of the threshold utility. A benefit to the simple satisficing model is that it can be run on standard data, using, for example, the same assumptions as in Sandorf and Campbell [8], but is best suited when evaluation order is known. For this reason, we also test the model on stated choice experiment data designed to compare the standard way of displaying all alternatives at the same time to one in which respondents actively choose between revealing another alternative or select their preferred alternative from those they have already revealed. Our results show that our model nests a “choose the first alternative” strategy, which is analogous to the no deliberation strategy in Manski [10], and a utility maximization strategy, which is analogous to the optimization strategy in Manski [10]. We discuss the implications of our results for welfare analysis and prediction.

## Econometric approach

To introduce notation, we assume that a decision maker faces a choice between *J* different alternatives provided in the complete and exhaustive choice set *C*_*n*_. Decision makers are indexed by *n* ∈ {1, 2, …, *N*} and alternatives by *j* ∈ {1, 2, …, *J*}. The utility, denoted by *u*, decision maker *n* receives from choosing the *j*^th^ alternative can be described by the random utility function in Eq 1.

$$\begin{array}{c} u_{nj}=v_{nj}+\varepsilon_{nj}=\beta x_{nj}+\varepsilon_{nj}, \end{array}$$ (1)

where **x**_*nj*_ is a column *k*-vector of the attributes of alternative *j* encountered by individual *n*, **β** is a conformable row *k*-vector of unknown marginal utility parameters to be estimated and *ε*_*nj*_ is an idiosyncratic random disturbance term. The observable part of utility (i.e., *v*_*nj*_) is made up of **βx**_*nj*_ and the unobservable part of utility is made up of *ε*_*nj*_. Under the assumption that *ε*_*nj*_ is a deviate from an identically and independently (*i.i.d.*) extreme value distribution with variance *π*^2^/6, the probability that alternative *j* is chosen by individual *n* can be represented by a conditional logit model [24, 25]:

$$\begin{array}{c} \text{Pr}{\left(j_{n}|X_{n},\beta \right)}_{\text{MNL}}=\frac{\text{exp}(\beta x_{nj})}{\sum_{j\in C_{n}}\text{exp}\left(\beta x_{nj}\right)}. \end{array}$$ (2)

All terms in Eq 2 are as defined above. The probability in Eq 2 follows from the classic utility maximization problem and we assume that the choice is made from the grand choice set of alternatives.

### Satisficing model

When people make choices, they do not always choose the utility maximizing alternative. One possibility is that they choose the first alternative exceeding some minimum level of acceptable utility. Let us define the minimum level of utility, or threshold utility, as *t*. The threshold utility is a function of the aspiration levels for each of the attributes making up an alternative. The threshold utility then is the (marginal) utility derived when all aspiration levels are met and as such is directly informed by a decision maker’s preferences. This then connects Selten’s [23] view of aspiration levels with our notion of a threshold utility and, furthermore, provides a mechanism for how the threshold utility changes in response to revised aspiration levels [23]. For example, assume that you are in the market to buy a car. Furthermore, when it comes to cars, you only care about the engine size and fuel economy. The absolute minimum for buying a new car is that it is better than your current car. This implies that the aspiration levels are set to the levels of your current car and that the threshold utility is the utility derived from owning your current car. In other words, the threshold would be based on some kind of status-quo situation. If this was indeed the threshold and you were satisficing, you would buy the first car you see that is better than your car subject to your budget constraint. This is rather unlikely. It is more likely that you have a minimum set of aspiration levels, e.g., a minimum engine size and a minimum level of fuel economy. Once these are decided upon, the preferences for these aspiration levels lead to the derived threshold utility. The threshold utility then is fully determined by your preferences and aspiration levels.

Just as we cannot observe an individual’s utility function, we cannot observe their aspiration levels nor the resulting threshold utility. For this reason, and to avoid trying to determine each individual aspiration level, we make probabilistic statements about whether or not the utility of the alternative exceeds the threshold. Let us define the threshold *t*_*n*_ as being comprised of an observable component *τ* to be estimated and an unobservable component *ϵ*_*n*_, such that *t*_*n*_ = *τ* + *ϵ*_*n*_. We assume that *ϵ*_*n*_ is *i.i.d.* and follows an extreme value distribution and furthermore that the differences in the unobserved parts of *u*_*nj*_ and *t*_*n*_ (i.e., *ε*_*nj*_ and *ϵ*_*n*_, respectively) are logistically distributed with the scale parameter being unity. This implies that the threshold utility determining a satisfactory alternative is independent of the utility experienced from any given alternative. While this assumption may seem restrictive at first glance, we argue that it is, in fact, quite trivial. To see this, we need to emphasize that within a given decision context and decision maker, there is only one threshold utility and that this utility is fully determined by a given decision maker’s preferences. This follows directly from the discussion above relating to aspiration levels. To continue with our new car example, before you even begin looking at a new car, you have an idea about what would be satisfactory levels of engine size and fuel economy, i.e., you already have established aspiration levels and consequently a threshold utility for what would be an acceptable car fully informed by your preferences. When you encounter a new alternative, i.e., you look at a new car, the utility you derive is a function of that alternative’s attributes and determined by your preferences. The utility you derive from this new alternative is independent of previously seen alternatives and your threshold utility since all are determined by the same set of underlying preferences for the attributes comprising these alternatives. Note that this implicitly assumes that there is no preference learning as you view more cars. A point which we remark on again below.

Having established this, we can express the probability that alternative *j* yields utility greater than the threshold using a logistic function:

$$\begin{array}{c} \text{Pr}(u_{nj}>t_{n}|x_{nj},\beta ,\tau )=\text{Pr}(v_{nj}+\varepsilon_{nj}>\tau +\epsilon_{n})=\text{Pr}(\varepsilon_{nj}-\epsilon_{n}>\tau -v_{nj})\\ =\frac{1}{1+\text{exp}(\tau -\beta x_{nj})}. \end{array}$$ (3)

Inherent in the satisficing model is the assumption that decision makers consider alternatives sequentially. This opens up for the possibility that a decision maker revises the threshold as they progress through the search and evaluation process [1, 8, 23]. This adjustment of the threshold may reflect a learning process, either of preferences or the range of possible alternatives in the market. Let us continue with the car example. Before visiting dealerships, it is important to you that your next car has good fuel economy and a large engine, but that fuel economy is the most important. As you visit different dealerships, you realize that there is a trade-off between the two and that a larger engine usually comes at the cost of worse fuel economy. Consequently, you may adjust your aspiration level for engine size down [23] and it follows that threshold utility also adjusts down. Despite the importance of aspiration adaption [23] in satisficing models, to keep the model simple and tractable, we assume that the threshold is stable throughout the entire decision process. Obviously, extending the model in this direction would be interesting. One possibility would be to parameterize *τ* with specific (possibly individual level) aspiration levels such that the threshold utility *t*_*nt*_ would reflect changing aspiration as individuals progress through the sequence of alternatives. This also provides another way to think about the threshold utility function as it is specified here: It is simply estimated as a constant with none of the attributes specified meaning that it is a collective parameter that would capture overall changes in threshold utility without capturing individual changes in aspiration levels. Importantly, whether the aspiration levels are specified in the function does not affect the ability of the model to capture potential search costs, which can also be added to the threshold function.

Given the sequential manner in which decision makers consider alternatives, the probability of an alternative being chosen in a satisficing model must account for the probability that all subsequent alternatives were not chosen:

$$\begin{array}{c} \begin{array}{c} \text{Pr}(j_{n}|X_{n},\beta ,\tau ,\text{Satisficing})=\{\begin{array}{cc} \text{Pr}(u_{nj}>t_{n}|x_{nj},\beta ,\tau ) & \text{if}\;j=1\text{;}\;\text{or,}\\ \text{Pr}(u_{nj}>t_{n}|x_{nj},\beta ,\tau )\prod_{j\in \{1,\ldots ,j-1\}}(1-\text{Pr}(u_{nj}>t_{n}|x_{nj},\beta ,\tau )) & \text{if}\;j>1. \end{array} \end{array} \end{array}$$ (4)

The probability that none of the alternatives in the choice set yield utility that exceeds the threshold utility is simply one minus the sum of the probability of an alternative being chosen in a satisficing model over all alternatives:

$$\begin{array}{c} \text{Pr}(u_{n}<t_{n}|X_{n},\beta ,\tau )=1-\sum_{j\in C_{n}}\text{Pr}(j_{n}|X_{n},\beta ,\tau ,\text{Satisficing}), \end{array}$$ (5)

where 0 < Pr(**u**_*n*_ < *t*_*n*_ ∣ **X**_*n*_, **β**, *τ*) < 1.

Given the strict inequality Pr(**u**_*n*_ < *t*_*n*_ ∣ **X**_*n*_, **β**, *τ*) > 0, there remains a probability that the choice task contains no satisfactory alternative, meaning that, after evaluating all possible alternatives, a decision maker must switch to another, secondary, decision strategy. Therefore, Pr(**u**_*n*_ < *t*_*n*_ ∣ **X**_*n*_, **β**, *τ*) can be interpreted as the probability of decision maker *n* switching to a secondary decision rule after they have evaluated all *J* alternatives in choice set *C*_*n*_ and established that none of them meet their acceptable threshold utility. The overall choice probability then becomes the satisficing probability plus the choice probabilities derived conditional on the secondary decision rule weighted by the probability that this rule is enacted:

$$\begin{array}{c} \text{Pr}(j_{n}|X_{n},\beta ,\tau ,1^{\text{st}}:\text{Satisficing},2^{\text{nd}}:\cdot )=\text{Pr}(j_{n}|X_{n},\beta ,\tau ,\text{Satisficing})\\ +\text{Pr}(u_{n}<t_{n}|X_{n},\beta ,\tau )\text{Pr}(j_{n}|\cdot ), \end{array}$$ (6)

where 1^st^:Satisficing and 2^nd^:· signify the primary and secondary decision making rules, respectively, and Pr(*j*_*n*_ ∣ ⋅) is the probability of choice conditional on the secondary decision making strategy. The secondary decision rule may entail a combination of decision making strategies and possible heuristics. In this paper, we consider four such secondary, or backup, strategies: i) utility maximization; ii) choose the opt-out; iii) choose the last; and, iv) choose at random.

The first strategy is where the decision maker chooses the utility maximizing alternative, which is represented by a conditional logit expression:

$$\text{Pr}(j_{n}|{\text{X}}_{n},\beta ,\text{RUM})=\frac{\text{exp}(\beta {\text{x}}_{nj})}{\sum_{j\in C}\text{exp}(\beta {\text{x}}_{nj})}.$$ (7a)

Secondly, we consider the strategy where the decision maker chooses to opt-out or chooses the explicitly offered status-quo option:

$$\begin{array}{c} \text{Pr}(j_{n}|\text{Opt-out})=\{\begin{array}{cc} 1 & \text{if}\;j=\text{opt-out}\;\text{or}\;\text{status-quo;}\;\text{and,}\\ 0 & \text{otherwise}. \end{array} \end{array}$$ (7b)

The third strategy is the situation where the decision maker simply chooses the last alternative in the choice set. This may, for example, arise due to a lack of recall or because alternatives are only temporarily available (e.g., as in the case when choosing a car parking space [26]). The conditional probability of choice under this strategy is given by:

$$\begin{array}{c} \text{Pr}(j_{n}|\text{Last})=\{\begin{array}{cc} 1 & \text{if}\;j=J\text{;}\;\text{and,}\\ 0 & \text{otherwise}. \end{array} \end{array}$$ (7c)

Finally, we consider the strategy where the decision maker chooses a random alternative after establishing that none of the available alternatives provide utility greater than the threshold. This leads to the following conditional probability:

$$\begin{array}{c} \text{Pr}(j_{n}|\text{Random})=\frac{1}{J}. \end{array}$$ (7d)

### Properties of the model

The model outlined in Eq 6 benefits from nesting three different models. As the threshold, *τ*, goes to −∞ every single alternative will have a utility higher than the threshold (i.e., lim_*τ*→−∞_ Pr(**u**_*n*_ < *t*_*n*_ ∣ **X**_*n*_, **β**, *τ*) = 0). In this case, choosing the first alternative that exceeds the threshold involves choosing the first alternative you are presented with. If search costs are subtracted from the threshold utility, then this becomes analogous to the no deliberation strategy outlined by Manski [10]. Indeed, letting the threshold be a function of cost is an interesting (and simple) extension of the proposed model in the current paper. The choice probability under a no deliberation strategy is equal to 1 and will approach 1 under a satisficing strategy as *τ* goes to −∞. The consequence of this is that if the threshold utility is sufficiently low such that *τ* identifies every choice as a satisficing choice, then the log-likelihood value of the model will tend to zero. This is expected when the model perfectly describes the data generation process. Conversely, as *τ* goes to + ∞, none of the alternatives will give a utility that is higher than the threshold (i.e., lim_*τ*→+ ∞_ Pr(**u**_*n*_ < *t*_*n*_ ∣ **X**_*n*_, **β**, *τ*) = 1). The model will, therefore, collapse to the model associated with the secondary decision rule. In this case, the model has the same fit and retrieves the same parameters as the secondary model, but is less parsimonious.

While the probability of the threshold utility being higher than the utility of all alternatives in the choice set in Eq 5 is, appropriately, unaffected by the order in which alternatives are evaluated, the satisficing choice probability in Eq 4 and, thus, the joint choice probability in Eq 6 are affected. Therefore, the evaluation order must be known. In cases where this is not known, it will be necessary to rely on simplifying assumptions. For example, if the position of alternatives is known, one could assume that people process alternatives from left to right or top to bottom in a sequential manner [8, 27]. Of course, the suitability of this assumption is an empirical decision and should be considered on a case-by-case basis, requiring discretion and objective judgment on behalf of the analyst (see Sandorf and Campbell [8] for a discussion and Campbell and Erdem [28] and for a related discussion on the influence of position on information processing). It is also necessary to assume that people choose the first alternative exceeding their threshold utility. If they choose the second or third alternative exceeding the threshold, they cannot have chosen according to a satisficing decision rule.

Similarly, if opting-out is an option, or if there is an explicitly offered status-quo option, it will be necessary to make assumptions regarding the order in which the opt-out alternative is evaluated. If individuals consider it as a reference point it is effectively the first encountered alternative. In this case, opt-out or status-quo choices would be consistent with satisficing behavior in situations where individuals deem this option to be both satisfactory and sufficient and do not evaluate any of the non-opt-out or non-status-quo alternatives. Depending on the decision context this could be difficult to distinguish from not entering the market to begin with. Conversely, in situations where individuals evaluate options before choosing to opt-out or the status-quo alternative, it is effectively the final alternative in the choice task. Ultimately, this must be determined by the analyst.

A further aspect of the model is that the probability of switching to a secondary decision rule depends also on the number of alternatives in the choice set. As one would expect, as the choice set grows in terms of the number of alternatives the probability that the secondary decision rule is needed reduces. This comes directly from taking the product in calculating the satisficing probability in Eq 4. But more obviously, as the number of alternatives increases the likelihood of encountering a satisfactory alternative can also be expected to increase, all else being equal.

We assume that all individuals use satisficing as their primary decision making rule and that they use one of four decision rules as their secondary rule. Admittedly, this has the potential of predicting choice outcomes that may be at odds with outcomes driven by resource rational decision making. Furthermore, in reality, every individual will use a strategy (or combination of strategies) that may be unique to them and that is likely to be highly dependent on the choice context. Hence, the assumption of persistent use of the same primary and secondary rule is a simplification. This limitation could, of course, be potentially relaxed through the use of probabilistic decision rule process models that accommodate heterogeneity in decision making strategies across individuals (e.g., see Hensher et al. [29]). This form of model recognizes that an individual’s actual decision making process is unobserved and cannot be known with certainty, but probabilistic statements about the likelihood of competing decision strategies being their true strategy can be reached based on their observed choices. However, this goes beyond the aims and scope of the paper. The purpose of the current paper is to develop a simple and tractable model to capture satisficing behavior. Readers interested in an application of this type of model to systematically explore satisficing behavior are directed to Sandorf and Campbell [8].

We assume that the threshold utility is stable throughout the entire decision process. As discussed above, this assumption can be relaxed by parameterizing *τ* to reflect changing aspiration levels as individuals progress through the sequence of alternatives and choice tasks. Moreover, following Güth [30] and Güth et al. [31], there may be a desire to investigate if satisficing behavior is absorbable (i.e., whether individuals continue to use it as a decision rule when they become aware of it). It is, however, challenging to separately explore absorbability and threshold revision within the current framework because it is difficult to know whether changes in *τ* are driven by a change in decision rule once they are aware that they satisfice, i.e, *τ* goes to + ∞ to collapse to a secondary rule, or if it is because aspiration levels change in light of experience. While parameterizing *τ* will help get closer to separately identifying the two, confounding between the constants and the threshold parameters is likely to remain.

Related, for this paper, we assume a constant *τ*, which implies that everyone has the same observable threshold utility. A pure satisficing strategy lies where *τ* uniquely identifies all choices in the data. For obvious reasons, this may require *τ* to be individual-specific, and in many, but not all, settings it makes sense to set the threshold utility to be equal to or higher than the utility of the opt-out or status-quo alternative. As discussed above, to move away from the status-quo, the new situation has to be at least as good. To fully capture satisficing behavior, an easy extension to the model involves reparameterization of *τ* to accommodate the potential influence that individual ability, motivation and a range of other, perhaps unobserved, factors have on the likelihood to satisfice; or to fully specify the alternative in terms of aspiration levels to allow for changing threshold utilities through adapted aspiration levels. Of course, there is also scope for further specifications to accommodate preference heterogeneity. Indeed, this may be, in fact, a necessary step to, at least partially, alleviate potential confounding concerns between **β** and *τ*.

### Analytical example

To illustrate, in Table 1 we show how the choice probabilities of an alternative being chosen under satisficing are dependent on *τ*, evaluation order and the secondary decision making rule. For demonstration purposes, we assume *τ* ∈ {−3, 0, 3, 6}, there are five alternatives to choose from (i.e., *J* = 5), the observed utilities *v*_*j*_ ∈ {−2, −1, 0, 1, 2}, and that the alternatives are either evaluated from the one that provides the lowest observed utility to the one that provides the highest observed, or vice versa. For the case where the secondary decision rule is to choose the opt-out alternative, we show only the results for when it is assumed to be the first evaluated alternative since the results for when individuals evaluate all options before choosing the opt-out can be ascertained from the choose the last strategy.

**Table 1:** Choice probability of an alternative being chosen under satisficing (for different satisficing thresholds and evaluation order of alternatives).

*v*_*j*_	Alternatives evaluated from lowest to highest					Alternatives evaluated from highest to lowest				
	-2.000	-1.000	0.000	1.000	2.000	2.000	1.000	0.000	-1.000	-2.000
*τ* = -3.000 Pr(**u** < *t* ∣ **v**, *τ*) = 0.000										
Pr(*u*_*j*_ > *t* ∣ *v*_*j*_, *τ*)	0.731	0.881	0.953	0.982	0.993	0.993	0.982	0.953	0.881	0.731
Pr(*j* ∣ *v*_*j*_, *τ*, Satisficing)	0.731	0.237	0.031	0.001	0.000	0.993	0.007	0.000	0.000	0.000
Pr (*j* | *v*_*j*_, *τ*, 1^st^:Satisficing, 2^nd^:RUM	0.731	0.237	0.031	0.001	0.000	0.993	0.007	0.000	0.000	0.000
Pr (*j* | *v*_*j*_, *τ*, 1^st^:Satisficing, 2^nd^:Opt-out	0.731	0.237	0.031	0.001	0.000	0.993	0.007	0.000	0.000	0.000
Pr (*j* | *v*_*j*_, *τ*, 1^st^:Satisficing, 2^nd^:Last	0.731	0.237	0.031	0.001	0.000	0.993	0.007	0.000	0.000	0.000
Pr (*j* | *v*_*j*_, *τ*, 1^st^:Satisficing, 2^nd^:Random	0.731	0.237	0.031	0.001	0.000	0.993	0.007	0.000	0.000	0.000
*τ* = 0.000 Pr(**u** < *t* ∣ **v**, *τ*) = 0.010										
Pr(*u*_*j*_ > *t* ∣ *v*_*j*_, *τ*)	0.119	0.269	0.500	0.731	0.881	0.881	0.731	0.500	0.269	0.119
Pr(*j* ∣ *v*_*j*_, *τ*, Satisficing)	0.119	0.237	0.322	0.235	0.076	0.881	0.087	0.016	0.004	0.001
Pr (*j* | *v*_*j*_, *τ*, 1^st^:Satisficing, 2^nd^:RUM	0.119	0.237	0.323	0.238	0.083	0.887	0.090	0.017	0.005	0.002
Pr (*j* | *v*_*j*_, *τ*, 1^st^:Satisficing, 2^nd^:Opt-out	0.130	0.237	0.322	0.235	0.076	0.891	0.087	0.016	0.004	0.001
Pr (*j* | *v*_*j*_, *τ*, 1^st^:Satisficing, 2^nd^:Last	0.119	0.237	0.322	0.235	0.087	0.881	0.087	0.016	0.004	0.012
Pr (*j* | *v*_*j*_, *τ*, 1^st^:Satisficing, 2^nd^:Random	0.121	0.239	0.324	0.237	0.078	0.883	0.089	0.018	0.006	0.003
*τ* = 3.000 Pr(**u** < *t* ∣ **v**, *τ*) = 0.598										
Pr(*u*_*j*_ > *t* ∣ *v*_*j*_, *τ*)	0.007	0.018	0.047	0.119	0.269	0.269	0.119	0.047	0.018	0.007
Pr(*j* ∣ *v*_*j*_, *τ*, Satisficing)	0.007	0.018	0.046	0.111	0.220	0.269	0.087	0.031	0.011	0.004
Pr (*j* | *v*_*j*_, *τ*, 1^st^:Satisficing, 2^nd^:RUM	0.014	0.037	0.098	0.251	0.601	0.650	0.227	0.082	0.030	0.011
Pr (*j* | *v*_*j*_, *τ*, 1^st^:Satisficing, 2^nd^:Opt-out	0.605	0.018	0.046	0.111	0.220	0.867	0.087	0.031	0.011	0.004
Pr (*j* | *v*_*j*_, *τ*, 1^st^:Satisficing, 2^nd^:Last	0.007	0.018	0.046	0.111	0.818	0.269	0.087	0.031	0.011	0.602
Pr (*j* | *v*_*j*_, *τ*, 1^st^:Satisficing, 2^nd^:Random	0.126	0.138	0.166	0.230	0.340	0.389	0.207	0.150	0.131	0.124
*τ* = 6.000 Pr(**u** < *t* ∣ **v**, *τ*) = 0.972										
Pr(*u*_*j*_ > *t* ∣ *v*_*j*_, *τ*)	0.000	0.001	0.002	0.007	0.018	0.018	0.007	0.002	0.001	0.000
Pr(*j* ∣ *v*_*j*_, *τ*, Satisficing)	0.000	0.001	0.002	0.007	0.018	0.018	0.007	0.002	0.001	0.000
Pr (*j* | *v*_*j*_, *τ*, 1^st^:Satisficing, 2^nd^:RUM	0.012	0.032	0.086	0.234	0.636	0.636	0.234	0.086	0.032	0.012
Pr (*j* | *v*_*j*_, *τ*, 1^st^:Satisficing, 2^nd^:Opt-out	0.972	0.001	0.002	0.007	0.018	0.990	0.007	0.002	0.001	0.000
Pr (*j* | *v*_*j*_, *τ*, 1^st^:Satisficing, 2^nd^:Last	0.000	0.001	0.002	0.007	0.990	0.018	0.007	0.002	0.001	0.972
Pr (*j* | *v*_*j*_, *τ*, 1^st^:Satisficing, 2^nd^:Random	0.195	0.195	0.197	0.201	0.212	0.212	0.201	0.197	0.195	0.195

From Table 1, we see that irrespective of evaluation order, with relatively small thresholds (in this example where *τ* ≤ −3) there is practical certainty that the choice set contains an alternative that exceeds the utility threshold (i.e., Pr(**u** < *t* ∣ **v**, *τ*≤ −3) ≈ 0). Note also that with very small thresholds practically all of the choice probability is allocated to the first evaluated alternative. Whereas, with relatively high thresholds (in this case where *τ* ≥ 6) the probability that any alternative yields utility above the threshold is practically zero and, as a result, the contribution of the satisficing choice probabilities to the likelihood function is, in effect, zero (i.e., Pr(**u** < *t* ∣ **v**, *τ* ≥ 6) ≈ 1). It is important to point out that the non-infinite boundaries of *τ* that produce values of Pr(**u** < *t* ∣ **v**, *τ*) that are distinguishable from 0 and 1 depends entirely on the observed utilities assumed here. Indeed, the effect of *τ* on the satisficing choice probabilities depends on its relative magnitude to the observed utilities. This aside, we can see that *τ* affects the overall joint likelihoods under the settings used in this analytical demonstration. With relatively low values of *τ* we can see that the probability of requiring a secondary decision making strategy is very small. In such cases, the joint likelihoods for the four secondary strategies are, therefore, practically equivalent to the satisficing choice probabilities. However, as *τ* increases, the satisficing choice probabilities get smaller meaning that the choice shares for the secondary strategies make a larger contribution to the likelihood function. As *τ* approaches its infinite boundary, effectively all of the likelihood is explained by the backup decision rule.

Even though the full choice set comprises the same alternatives, Table 1 clearly shows that different evaluation sequences can lead to markedly different probabilities. This difference is most stark with relatively low values of *τ* (since more of the likelihood is explained by the satisficing choice probabilities). Take, for instance, the case where *τ* = −3 and focusing on the alternative with the highest observable utility of 2. When alternatives are evaluated from the one that provides the lowest utility to the highest utility, the respective joint choice probabilities are practically zero. However, in the case where the alternatives are evaluated in the opposite order, the respective joint choice probabilities are effectively one. In this example, not until *τ* ≥ 6, in which case the backup strategy essentially explains all of the likelihood function, do we find the joint probabilities to be relatively commensurate for the utility maximizing and random choice secondary strategies. For the choose opt-out and last alternative backup strategies, however, the probabilities are more commensurate at the non-infinite boundaries of *τ*, which is because sequence order plays an additional role in both strategies. Under the settings of this analytical example, *τ* ≈ 1.80 yields the most comparable probabilities for the alternative with the highest utility in these backup strategies. This is especially the case for choosing the last alternative secondary decision rule, where the probabilities are practically equivalent, albeit the probabilities for the other alternatives remain somewhat different.

## Synthetic application

### Data

To test the performance of our model and how well it retrieves the true parameters under varying experimental conditions we run a series of Monte-Carlo simulations. Our Monte-Carlo strategy involves a variety of generation processes. To test the ability of the model to correctly retrieve the parameters under designs with varying numbers of alternatives, we generate data where *J* ∈ {2, 3, 4, 5, 6, 8, 10, 25, 50} alternatives. Each alternative is described by four generic attributes: AttA and AttB, which have binary (0, 1) levels; AttC, which takes levels between 0 and 1 in 0.01 increments; and, Cost, which has levels between €5 and €30 in €0.50 increments. Thus, the full factorial consists of 20,604 profiles (i.e., two levels for AttA times two levels for AttB times 101 levels for AttC times 51 levels for Cost). We assume that the true parameters were: 0.5 for AttA, 0.8 for AttB, -1.6 for AttC, and -0.1 for Cost, and that the alternative-specific constants are all zero.

We generate data based on different assumptions regarding the level of the threshold utility. The threshold utilities are derived by generating the full factorial design, which consists of all possible combinations of the levels of the attributes, and for each profile generate Eq 1 based on 1,000 simulated draws of *ε* per profile. We then derive the minimum, maximum and intermediate ventile utility values for the simulated full factorial design, thus producing 21 values of *τ*, which are reported in Table 2. This leads to 189 (i.e., nine settings relating to the number of alternatives times 21 settings relating to *τ*) different simulation treatments. Each treatment consists of 1,000 individuals answering a single choice task. We note that a panel of repeated choice contexts could also be accommodated under this framework. However, in this data generation process, we assume preference homogeneity and a constant threshold meaning that having a panel would be redundant. Though it is recognized that the ability to identify satisficing behavior and the threshold employed by a given individual will be higher in panel data since a behavioral rule that is respected over a sequence of multiple choices is, clearly, more convincing than one observed in a single choice (see Sandorf and Campbell [8] for an exploration of this issue). The experimental design for each simulated dataset was generated at random. Since idiosyncratic results can arise from a single sample, we generate multiple replications of the experimental design. In total, we generate 1,000 replications for the 189 treatments.

**Table 2:** Threshold utilities.

%ile	0.00	0.05	0.10	0.15	0.20	0.25	0.30	0.35	0.40	0.45	0.50	0.55	0.60	0.65	0.70	0.75	0.80	0.85	0.90	0.95	1.00
*τ*	-7.74	-3.76	-3.29	-2.96	-2.69	-2.45	-2.23	-2.03	-1.83	-1.64	-1.44	-1.25	-1.04	-0.83	-0.60	-0.35	-0.06	0.30	0.77	1.52	23.83

The individual counterfactual choices are produced by identifying the first alternative where *u*_*nj*_ ≥ *t*. If *u*_*nj*_ < *t*∀*j* the choices are determined based on the four models in Eq 7. In this case, this, respectively, involves identifying the alternative with the largest utility value, the first (i.e., opt-out) alternative, the last alternative, or a random alternative.

### Analysis

For every dataset generated, we estimate two candidate models: (i) the naïve specification based solely on the respective secondary decision rule (where we retrieve parameter estimates for the marginal utilities and alternative-specific constants for the first and last alternatives); and, (ii) the specification where satisficing is used as the primary decision rule and the respective strategy as the secondary decision rule (where we, again, retrieve parameter estimates for the marginal utilities and alternative-specific constants for the first and last alternatives in addition to the threshold utility). Strictly speaking, the naïvely specified model is only estimated for the treatment where the utility maximizing alternative is chosen as the backup strategy. For the other secondary decision rule settings, the shares are conditional only on the data and can be established deterministically since they are equal to the sample shares for the respective decision rule. We retrieve alternative-specific constants for the first and last alternatives to shed light on the potential misinterpretation of alternative-specific constants under satisficing behavior. We omitted these constants from the expressions in Section to avoid cluttering.

Estimating both candidate models allows us to compare the effects under correctly specified and misspecified cases and to make inferences regarding the consequences of the naïve assumption. Combined, this leads to a total of 1,512,000 (i.e., 189 simulation treatments times 1,000 replications times four secondary decision rules times two model specifications) models to estimate.

### Results

#### Observed choice shares by decision rule

**Fig 1:**
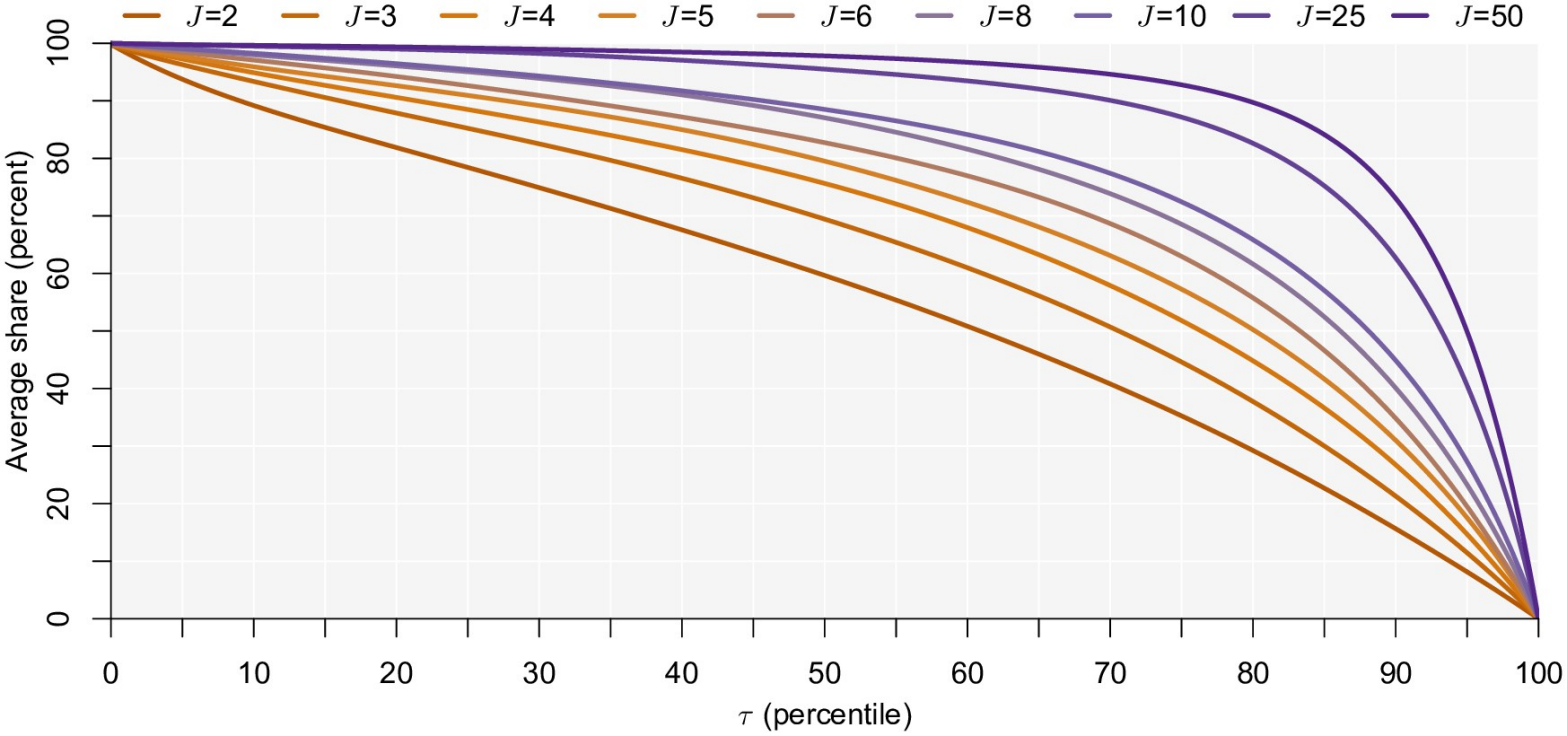
Share of choices consistent with satisficing.

In Fig 1, we compare the average share (across the 1,000 sample simulations) of simulated choices that are consistent with satisficing. We see that if the threshold is very low, practically all choices are consistent with satisficing. This is quite logical. With a sufficiently low threshold, any alternative encountered would be better and should be chosen under satisficing. As the threshold utility increases, the probability of satisficing decreases. If none of the alternatives meets the satisficing threshold, then the decision maker has to revert to their secondary decision rule. Crucially, the probability of satisficing depends on the number of available alternatives. We see that even at higher utility thresholds, the probability of satisficing remains high when many alternatives exist. Again, this is quite logical. If you can search through many alternatives, chances are higher that at least one of them will exceed the threshold. However, for sufficiently high thresholds, this probability drops rapidly to zero.

For the sake of brevity, the observed share for the secondary decision strategies as a function of the level of the threshold are presented and discussed in Appendix A in S1 Appendix. We observe that the share of choices that are consistent with the secondary decision rule increases with the threshold, which follows the same logic as above. This is found for all secondary decision rules apart from choosing the opt-out alternative, where a u-shaped pattern is observed because the opt-out is the first alternative encountered in our case. Across all decision rules, the share of choices consistent with the backup strategy reduces as the number of alternatives increases.

#### Correctly predicted

**Fig 2:**
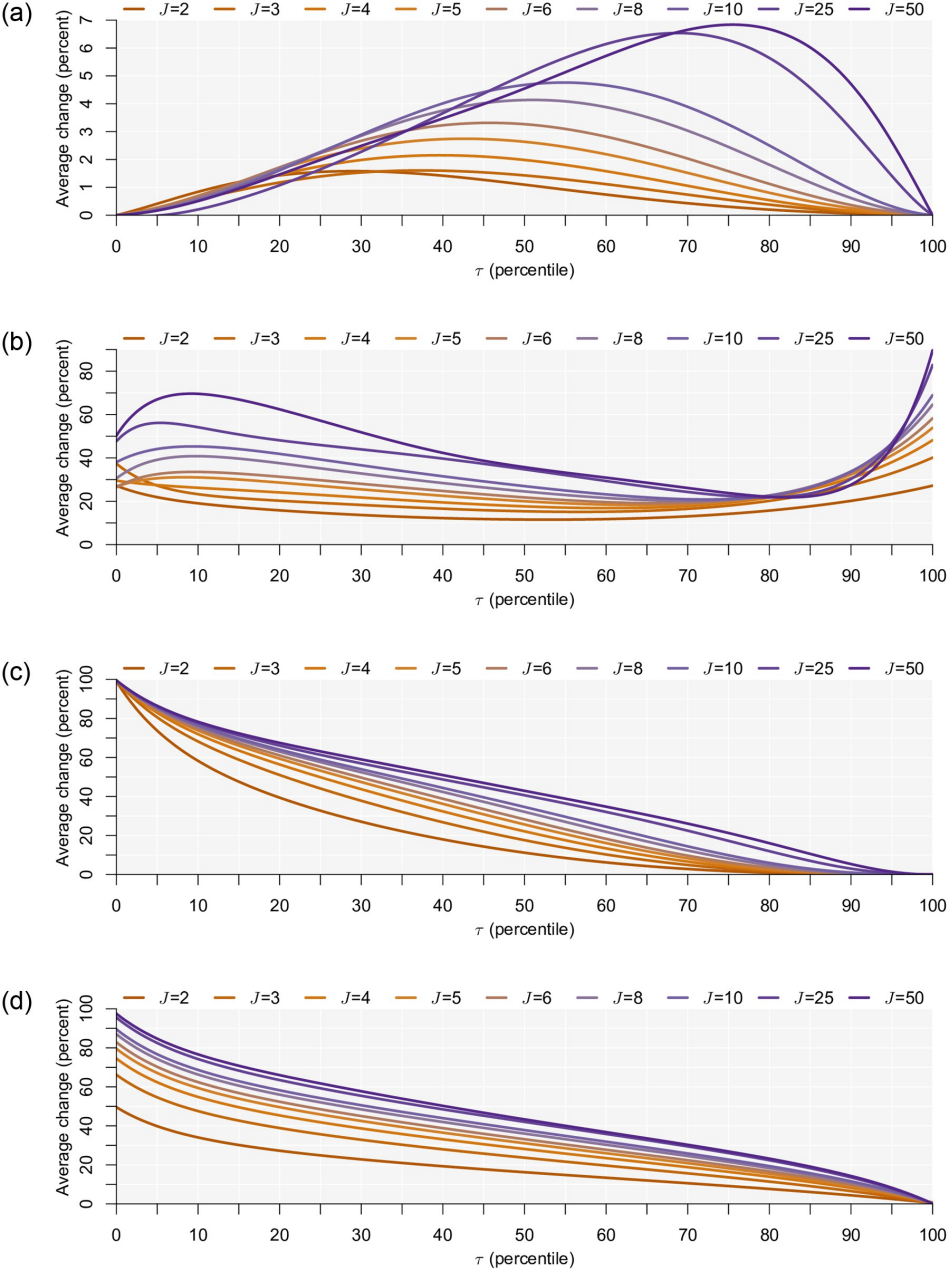
Change in choices correctly predicted under satisficing model relative to the naïve baseline model. **(a)** Data generated on basis of choose the utility maximizing alternative secondary decision rule. **(b)** Data generated on basis of choose the opt-out alternative secondary decision rule. **(c)** Data generated on basis of choose the last alternative secondary decision rule. **(d)** Data generated on basis of choose a random alternative secondary decision rule.

Since comparisons of model fit are possible for only the treatments where the utility maximizing alternative is chosen as the backup strategy (i.e., because the shares under the naïve models are established deterministically for the other secondary decision rules), we compare the percentage of choices that are correctly predicted as having the largest choice probability [32]. The weakness of this as a measure of goodness to fit is acknowledged—see Train [33] (page 69)—but is chosen as a way to allow more direct comparison across treatments. In Fig 2, we show the average (across the 1,000 sample simulations) percentage difference in correctly predicted, with the share observed for naïve specification being the subtrahend, broken down by the secondary decision rule.

Looking firstly at the results for the utility maximizing treatment in Fig 2a, the difference between the correctly predicted choices under the two candidate models is negligible at the infinite boundaries of *τ*. But note, this result for the lower infinite boundary is driven by the alternative-specific constant for the first alternative, which ensures the model fits are equivalent. Recall that the alternative-specific constants were zero in the data generation process, meaning that, while both models describe choices equally well at the lower extreme of *τ*, the naïve baseline model leads to biased behavioral inferences. As the threshold increases, the probability that this is true is decreasing. This stems from the fact that the model fits, and hence share of correctly predicted, of both models converge as the threshold increases. Of course, the satisficing model is less parsimonious with one additional parameter meaning that the gains achieved under the satisficing model will eventually be outweighed by the loss of parsimony. We note that the speed at which the models converge depends on the number of alternatives. Specifically, we see that as the number of alternatives grows, the increase in the share of choice correctly predicted peaks higher for higher thresholds. Thus, the implications of not considering satisficing behavior may be greater with larger choice sets even when the threshold utility is relatively high. This mirrors closely the results found above and the logic for why this is true is the same: as the number of alternatives grows, the probability that one of them will be satisfactory is also increasing. All this aside, we emphasize that the average increase in choices correctly predicted is relatively modest, at the maximum ranging between 1.78 percent and 7.60 percent for the case where *J* = 2 and *J* = 50, respectively. Looking at how this equates in terms of improvement in model fit, we find that the maximum average increase in model fit of the satisficing model ranges between around 25 and 760 log-likelihood units for *J* = 2 and *J* = 50, respectively. While both represent an improvement in model fit even after accounting for the loss of parsimony caused by the estimation of an additional parameter, it equates to an average increase of the choice probabilities of between just 1.6 and 7.8 percent, respectively. Thus, accounting for satisficing is unlikely to yield any substantial gains in model fit. Of course, model fit is not the only factor to consider as not accounting for satisficing may have implications for key behavioral outputs.

For choosing the opt-out alternative treatment in Fig 2b the satisficing model explains choices much better, especially so as the number of alternatives increases and at extreme values of *τ*. This latter observation stems from the fact that at both extremes more of the choice probability is allocated to the opt-out alternative because the first alternative exceeds the acceptable utility in the former and because none of the alternatives exceeds this threshold in the latter. In Fig 2c the naïve model assigns zero probability to the chosen alternative, whereas the satisficing model with choosing the opt-out alternative secondary decision rule predicts them perfectly. But the difference in correctly predicted drops as the value of *τ* increases, such that both models predict the last alternative being chosen. As *τ* approaches its upper infinite boundary this difference approaches zero. This is the case regardless of the number of alternatives, but *τ* approaches its upper infinite boundary sooner when there are fewer alternatives. In Fig 2d, a similar pattern to Fig 2a is observed. With a random choice secondary decision rule, the naïve model assigns 1/*J* to all chosen alternatives. At the lower infinite boundary of *τ*, where the first presented alternative is the chosen one, the satisficing model predicts the choice perfectly producing a difference in the likelihood of 1 − 1/*J*. However, as the threshold increases, more weight is allocated to the secondary decision rule resulting in a smaller difference in the share of choices correctly predicted.

#### Retrieving the true parameters

In addition to choice prediction, it is important to assess if the threshold utility *τ* is retrieved well. We use the root-mean-square errors (RMSEs) as indicators of our model’s ability to retrieve the true parameters. The RMSE is a measure of the magnitude of the difference between the estimated parameters and the true parameters used in the data generating process. It represents the standard deviation of the difference between predicted and actual values over the 1,000 replications, thus giving a single measure of the predictive power for a parameter of interest for all candidate models.

**Fig 3:**
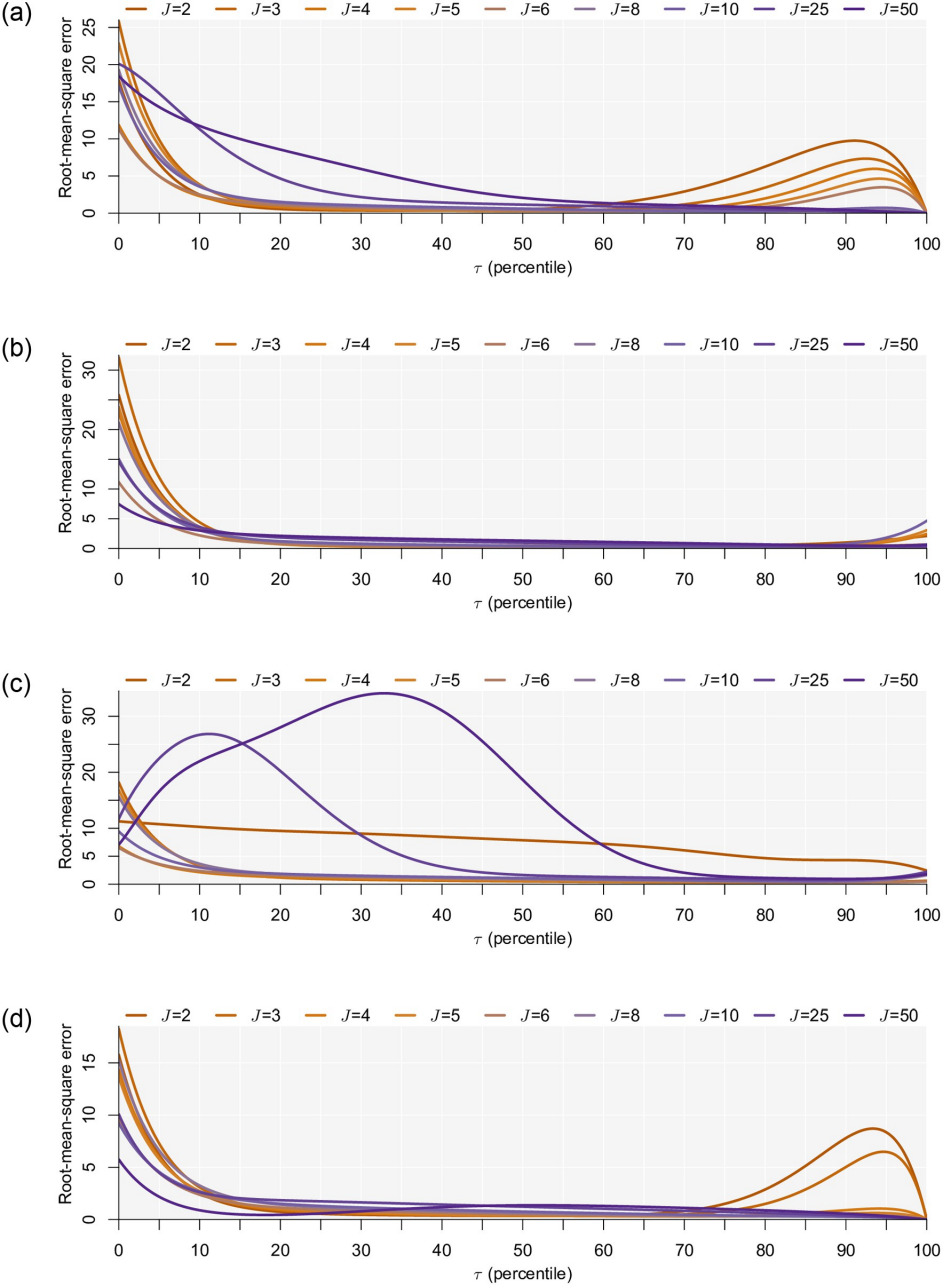
Accuracy of $\hat{\tau }$. **(a)** Data generated on basis of choose the utility maximizing alternative secondary decision rule. **(b)** Data generated on basis of choose the opt-out alternative secondary decision rule. **(c)** Data generated on basis of choose the last alternative secondary decision rule. **(d)** Data generated on basis of choose a random alternative secondary decision rule.

In Fig 3, we plot the RMSE share of estimated values of *τ*. For the data generated assuming the utility maximization secondary decision rule the plot in Fig 3a reveals that the threshold utility parameter is estimated relatively well for non-extreme threshold values. Thinking about the evidence above, this is, in fact, the region where we expect our satisficing model to work well. With a very low threshold, chances are very high that the utility of the first encountered alternative is higher. As such, it does not matter what value *τ* takes, the predicted probability is the same. The same argument holds for very large values of *τ* since any value of *τ* above this point will, for all intents and purposes, lead to the same predicted probabilites. Fig 3 appears to contradict this, but note that all models used the data generation parameters as starting parameters. As the threshold increases, the value of *τ* moves less from its original position. So, our model is only able to correctly identify the threshold parameter within a reasonable region. This result does follow from the properties of our model and mirrors the results of Manski [10]. Finally, we note that our ability to correctly identify the threshold parameter is dependent on the number of alternatives. In particular, as the number of alternatives increases a higher threshold is generally required to estimate it correctly. This suggests there to be a “Goldilocks” number of alternatives for this particular satisficing model (and maybe for satisficing models in general). It is clear that with too few alternatives, we tend to estimate the threshold better when it is relatively low, and with too many alternatives, we tend to estimate it better as it increases. Under the assumptions in this paper (and specific parameters of the data generating process), the threshold utility is generally best estimated (over the threshold range) when there are either five or six alternatives. The intuition is quite clear. With fewer alternatives, there is a reduced chance of seeing alternatives that exceed the threshold, and with many you are practically guaranteed to see one that exceeds the threshold. Given the probabilistic nature of our model, an alternative that yields utility that is minisculely smaller than the threshold leads to a probability of 0.5 in Eq 3. This could be what leads to the observed pattern. We do remark that more research is needed before this result can be generalized.

## Empirical application

### Data

Our experimental design aims to overcome the inherent limitation in the standard way of displaying alternatives in a stated choice experiment. We propose an approach that involves respondents actively revealing alternatives. This mimics a real world search process. We had to find a good that is relatively cheap so that people buy it with some frequency, yet rich enough in attributes to induce search. In this experiment, we use a decision maker’s choice among bottles of wine. The choice of wine is likely to capture both those who search for a particular bottle of wine that match their preferences and those who do not.

To decide on which attributes to include in our experiment, we relied on the information displayed on the shelves in the supermarket, information commonly displayed on the supermarket’s websites, attributes discussed on wine review websites and feedback from our informal focus groups. Based on this, we selected seven attributes: 1) country of origin, 2) color of the wine, 3) alcohol by volume, 4) grape variety, 5) characteristic of the wine, 6) whether the wine was organic, and 7) price. Next, we scraped the websites of three large supermarket chains in the UK to get the attribute information for all the wines they sell. We limited the levels of the “country of origin” and “grape variety” attributes to include only the eight most common countries and ten most common grape varieties (determined based on the results from the web-scraping). We limited the “characteristic of the wine” attribute to be on five-point scales ranging from dry to sweet for white and rosé wines and light to full-bodied for red wines, and we limited price to include 35 levels at varying increments. The full factorial included 1,848,000 possible combinations of our attributes. We excluded all infeasible combinations, e.g., grape variety and color, and characteristic of the wine and grape variety, which left us with 381,920 possible wines. Using the web-scraped wine data, we calculated a set of probability weights to establish the likelihood of each experimentally designed wine bottle being available in the supermarket. We used these weights to sample individual random profiles from the restricted factorial each time an individual entered the survey. The idea was that the alternatives available to a respondent would mimic (probabilistically) the wine selection in the supermarket and that the respondent will see a larger proportion of more familiar and likely wines as opposed to more unfamiliar and unlikely wines. Importantly, this random experimental design process assures that we have a lot of variation in our data, meaning that we eliminate order and path dependency in choices between respondents, such that any behavioral or choice patterns we detect are unlikely to be an artifact of the experimental design. Furthermore, given that respondents reveal alternatives sequentially, not all alternatives allocated to the individual design will be seen and considered by the respondent. Using a random design ensures that our ability to make inferences about the parameters is not systematically affected by respondents not revealing all alternatives. Lastly, the search path is also varied randomly but remains observed by the analyst, which should average out any path-dependency effects that might exist in the real world. As such our experimental data should cleanly predict behavior and provide testable hypotheses for real world data.

The survey was programmed in Shiny [34], which is an R package, and the data was gathered at the end of January 2020. In total, 4,121 respondents were randomly allocated to one of 10 treatments, each designed to test a specific aspect of search and preference learning. In the present paper, we rely on the first four of these:

- Treatment 1: Standard stated choice experiment with three alternatives and a “buy none” and comprises 554 individuals who, combined, made 4,774 choices.
- Treatment 2: Standard stated choice experiment with six alternatives and a “buy none” and comprises 541 individuals who, combined, made 4,652 choices.
- Treatment 3: Standard stated choice experiment with nine alternatives and a “buy none” and comprises 518 individuals who, combined, made 4,437 choices.
- Treatment 4: Sequential search stated choice experiment where a respondent could reveal up to nine alternatives by clicking a button and comprises 424 individuals who, combined, made 3,691 choices.

Our experimental setup falls under what Artinger et al. [35] would classify as satisficing under risk, where an individual is unaware of the available alternatives but has some information about their distribution, e.g., prior knowledge about types of wines or ranges of the attribute levels, or how costly it is to search (in our search treatment (i.e., treatment 4), the search cost was very close to zero). It is possible to work out what is the optimal choice, and, indeed, in our search treatment, a satisficing decision maker will choose the first alternative exceeding the utility threshold, however, a non-satisficing decision maker can choose among all revealed alternatives, i.e., perfect recall and availability. Regardless, there is a risk that the chosen alternative is suboptimal in the sense that there might be a better unrevealed alternative out there.

To show respondents how to answer the choice tasks, we created short instructional videos. The information was presented neutrally and was consistent across all treatments. To achieve this we created the videos using screen capture software and used Amazon Polly from AWS to create the voice-over. Respondents were instructed to watch the video carefully before proceeding with the choice tasks. Example tasks and the instruction videos can be found at https://choice-tasks.inspire-project.info/.

Respondents were randomly recruited from the UK population aged 18 and over. We did not use any quota sampling to ensure that our samples were representative of the target population. Our data collection effort was approved by the General University Ethics Panel of the University of Stirling (#: GUEP611). All respondents received an information sheet and were asked to complete an online consent form. The consent form asked respondents to tick a series of boxes to indicate that they understood the information provided to them and that they consented to participate. Only respondents who consented entered the survey.

### Results

#### Observed choice shares

Before continuing with the estimation results, we first consider the observed choice shares by alternative for the four treatments in Table 3. This reveals that there is a general downward trend in the share of choices as we move from the leftmost alternative (i.e., *j* = 1) to the rightmost alternative (i.e., *j* = 4, *j* = 7 and *j* = 10 in treatments 1, 2 and 3 as well as 4, respectively). While clearly not unequivocal evidence, it signals that there may be an inherent tendency to process the alternatives from left to right and to choose accordingly. This decline in choice share appears to get progressively more pronounced as the number of alternatives grows. In treatment 4, where the information search was controlled, the downward trend is especially stark, since the share of times each subsequent alternative was revealed (and thus part of the presented choice task) dropped (see final column). Indeed, the final alternative was revealed in less than 10 percent of cases (incidentally, this was mainly in the first choice task). Inspecting further, we find that in almost 60 percent of all choices respondents choose their last revealed alternative where additional alternatives were not revealed. On the face of it, this choice behavior is consistent with satisficing. However, upon further inspection, it is found that in just over 50 percent of these cases the status-quo alternative was chosen without revealing any non-status-quo alternatives. This makes it difficult to say for sure if a satisficing decision rule was adopted or if these choices are an artifact of some form of status-quo effect. Indeed, almost 25 percent of respondents in treatment 4 always choose the status-quo alternative (which is considerably higher compared to treatments 1, 2 and 3) and 20 percent never revealed another alternative. In any case, this still leaves over one-quarter of all choices in treatment 4 to be the respondent’s last revealed alternative where it is not the status-quo or final alternative.

**Table 3:** Choice breakdown by alternative.

*j*	Choice shares (percent)				Revealed share (percent)			
	Treatment 1	Treatment 2	Treatment 3	Treatment 4	Treatment 1	Treatment 2	Treatment 3	Treatment 4
1	28.84	22.03	19.65	45.38	100.00	100.00	100.00	100.00
2	23.48	13.54	12.10	18.67	100.00	100.00	100.00	69.47
3	26.90	16.85	14.24	10.00	100.00	100.00	100.00	53.05
4	20.78	16.04	12.40	8.45	100.00	100.00	100.00	46.74
5		12.64	10.25	6.58		100.00	100.00	39.39
6		10.08	7.66	4.25		100.00	100.00	29.29
7		8.81	6.33	2.76		100.00	100.00	21.70
8			6.13	1.65			100.00	15.55
9			6.18	1.35			100.00	11.08
10			5.05	0.89			100.00	8.18
Always *j* = 1	12.09	9.43	9.07	22.41	0.00	0.00	0.00	18.16

#### Estimation results

For the empirical data, we focus only on the utility maximization case and estimate two naïve specifications where it is the only decision rule (which we label RUM-OptOut and RUM-ASCs) and two specifications where it is the secondary decision rule enacted only if none of the alternatives meets the satisficing threshold (which we label SAT-OptOut and SAT-ASCs). The difference between the specifications is that in RUM-OptOut and SAT-OptOut, we only estimate an alternative-specific constant for the ‘none-of-these-bottles’ alternative and in RUM-ASCs and SAT-ASCs, we estimate the full *J* − 1 alternative-specific constants relative to the ‘none-of-these-bottles’ alternative. Results for treatments 1, 2, 3 and 4 are presented in Tables 4–7, respectively.

**Table 4:** Estimation results for treatment 1.

	RUM-OptOut	SAT-OptOut	RUM-ASCs	SAT-ASCs
Price	-0.087** (0.007)	-0.090** (0.007)	-0.087** (0.007)	-0.090** (0.008)
Country of origin				
Chile	-0.039 (0.095)	-0.051 (0.100)	-0.039 (0.095)	-0.048 (0.100)
France	0.041 (0.065)	0.046 (0.068)	0.039 (0.065)	0.044 (0.069)
Italy	0.009 (0.073)	0.010 (0.076)	0.010 (0.074)	0.011 (0.076)
New Zealand	-0.108 (0.094)	-0.112 (0.098)	-0.104 (0.095)	-0.105 (0.098)
South Africa	-0.182* (0.090)	-0.183 (0.093)	-0.183* (0.090)	-0.182 (0.093)
Spain	0.019 (0.075)	0.024 (0.078)	0.011 (0.075)	0.016 (0.079)
USA	-0.290** (0.078)	-0.299** (0.081)	-0.294** (0.078)	-0.303** (0.081)
Grape				
Cabernet Sauvignon	0.249** (0.089)	0.259** (0.094)	0.249** (0.090)	0.260** (0.095)
Chardonnay	0.268** (0.097)	0.277** (0.103)	0.271** (0.098)	0.279** (0.103)
Chenin Blanc	0.061 (0.085)	0.056 (0.088)	0.056 (0.086)	0.053 (0.089)
Malbec	0.146 (0.091)	0.145 (0.095)	0.145 (0.092)	0.147 (0.095)
Merlot	0.310** (0.084)	0.315** (0.088)	0.301** (0.085)	0.308** (0.088)
Pinot Grigio	0.310** (0.089)	0.320** (0.094)	0.309** (0.089)	0.317** (0.093)
Pinot Noir	0.219** (0.076)	0.224** (0.079)	0.222** (0.076)	0.228** (0.079)
Sauvignon Blanc	0.242* (0.102)	0.242* (0.107)	0.251* (0.103)	0.254* (0.107)
Tempranillo	0.080 (0.090)	0.082 (0.094)	0.073 (0.090)	0.074 (0.093)
Character				
Red wine	0.028 (0.019)	0.029 (0.020)	0.030 (0.019)	0.030 (0.020)
White wine	0.116** (0.023)	0.123** (0.024)	0.119** (0.023)	0.124** (0.025)
Organic	0.075 (0.043)	0.078 (0.045)	0.071 (0.043)	0.072 (0.045)
Alcohol by volume	0.032 (0.021)	0.033 (0.022)	0.032 (0.021)	0.034 (0.022)
ASC				
*j* = 1	0.255 (0.283)	0.166 (0.296)		
*j* = 2			-0.277 (0.284)	-0.242 (0.294)
*j* = 3			-0.125 (0.286)	-0.040 (0.304)
*j* = 4			-0.393 (0.285)	-0.272 (0.312)
*τ*		2.131** (0.373)		2.043** (0.553)
Log-likelihood	-6,371.815	-6,368.449	-6,352.218	-6,351.631
Observations	4,774	4,774	4,774	4,774
*K*	22	23	24	25
${\bar{\rho }}^{2}$	0.034	0.034	0.037	0.036
AIC	12,787.631	12,782.899	12,752.436	12,753.261
BIC	12,929.991	12,931.731	12,907.739	12,915.035

**Table 5:** Estimation results for treatment 2.

	RUM-OptOut	SAT-OptOut	RUM-ASCs	SAT-ASCs
Price	-0.084** (0.008)	-0.089** (0.008)	-0.086** (0.008)	-0.088** (0.008)
Country of origin				
Chile	0.011 (0.088)	-0.001 (0.094)	0.019 (0.088)	0.011 (0.091)
France	0.109 (0.062)	0.124 (0.066)	0.113 (0.062)	0.122 (0.066)
Italy	0.105 (0.071)	0.111 (0.076)	0.106 (0.071)	0.112 (0.075)
New Zealand	0.158* (0.080)	0.173* (0.085)	0.166* (0.081)	0.168* (0.083)
South Africa	-0.007 (0.079)	0.005 (0.084)	0.000 (0.078)	0.005 (0.082)
Spain	0.074 (0.071)	0.065 (0.076)	0.080 (0.071)	0.075 (0.073)
USA	-0.084 (0.080)	-0.094 (0.085)	-0.077 (0.080)	-0.085 (0.083)
Grape				
Cabernet Sauvignon	0.156 (0.080)	0.191* (0.086)	0.162* (0.081)	0.178* (0.085)
Chardonnay	0.120 (0.096)	0.113 (0.102)	0.121 (0.096)	0.121 (0.099)
Chenin Blanc	0.105 (0.085)	0.116 (0.090)	0.100 (0.085)	0.112 (0.089)
Malbec	0.181* (0.085)	0.212* (0.092)	0.201* (0.086)	0.214* (0.090)
Merlot	0.179* (0.076)	0.209* (0.082)	0.183* (0.076)	0.196* (0.080)
Pinot Grigio	0.431** (0.090)	0.459** (0.098)	0.429** (0.091)	0.449** (0.095)
Pinot Noir	0.006 (0.069)	0.024 (0.073)	0.014 (0.069)	0.022 (0.072)
Sauvignon Blanc	0.308** (0.091)	0.324** (0.099)	0.306** (0.091)	0.315** (0.095)
Tempranillo	0.064 (0.079)	0.071 (0.084)	0.077 (0.080)	0.080 (0.082)
Character				
Red wine	0.055* (0.022)	0.054* (0.023)	0.054* (0.022)	0.054* (0.023)
White wine	0.116** (0.023)	0.123** (0.025)	0.116** (0.023)	0.120** (0.024)
Organic	0.053 (0.042)	0.063 (0.044)	0.054 (0.042)	0.059 (0.044)
Alcohol by volume	0.013 (0.021)	0.012 (0.022)	0.014 (0.021)	0.013 (0.022)
ASC				
*j* = 1	0.465 (0.297)	0.086 (0.319)		
*j* = 2			-0.432 (0.302)	-0.370 (0.314)
*j* = 3			-0.207 (0.303)	-0.084 (0.322)
*j* = 4			-0.254 (0.303)	-0.074 (0.334)
*j* = 5			-0.506 (0.304)	-0.286 (0.345)
*j* = 6			-0.741* (0.305)	-0.489 (0.356)
*j* = 7			-0.876** (0.303)	-0.593 (0.365)
*τ*		1.710** (0.320)		1.990** (0.343)
Log-likelihood	-8,702.515	-8,645.718	-8,604.934	-8,603.495
Observations	4,652	4,652	4,652	4,652
*K*	22	23	27	28
${\bar{\rho }}^{2}$	0.036	0.042	0.046	0.046
AIC	17,449.030	17,337.437	17,263.868	17,262.990
BIC	17,590.821	17,485.673	17,437.885	17,443.452

**Table 6:** Estimation results for treatment 3.

	RUM-OptOut	SAT-OptOut	RUM-ASCs	SAT-ASCs
Price	-0.083** (0.008)	-0.085** (0.009)	-0.084** (0.009)	-0.084** (0.009)
Country of origin				
Chile	-0.048 (0.087)	-0.063 (0.093)	-0.060 (0.089)	-0.061 (0.089)
France	-0.026 (0.063)	-0.060 (0.070)	-0.038 (0.064)	-0.037 (0.065)
Italy	-0.046 (0.068)	-0.052 (0.076)	-0.057 (0.070)	-0.058 (0.070)
New Zealand	-0.014 (0.086)	0.011 (0.095)	-0.010 (0.088)	-0.010 (0.088)
South Africa	-0.079 (0.080)	-0.075 (0.086)	-0.093 (0.082)	-0.093 (0.082)
Spain	-0.050 (0.066)	-0.050 (0.073)	-0.051 (0.068)	-0.050 (0.068)
USA	-0.304** (0.075)	-0.354** (0.080)	-0.327** (0.077)	-0.326** (0.077)
Grape				
Cabernet Sauvignon	0.436** (0.080)	0.472** (0.084)	0.440** (0.081)	0.439** (0.081)
Chardonnay	0.242** (0.093)	0.245* (0.100)	0.253** (0.094)	0.252** (0.094)
Chenin Blanc	0.175 (0.091)	0.169 (0.097)	0.181 (0.093)	0.181 (0.093)
Malbec	0.222** (0.082)	0.272** (0.088)	0.244** (0.084)	0.243** (0.084)
Merlot	0.197* (0.083)	0.213* (0.087)	0.201* (0.084)	0.202* (0.084)
Pinot Grigio	0.409** (0.089)	0.429** (0.096)	0.428** (0.091)	0.427** (0.091)
Pinot Noir	0.242** (0.071)	0.279** (0.077)	0.244** (0.073)	0.245** (0.073)
Sauvignon Blanc	0.346** (0.094)	0.363** (0.102)	0.346** (0.097)	0.347** (0.097)
Tempranillo	0.285** (0.082)	0.344** (0.086)	0.299** (0.082)	0.299** (0.082)
Character				
Red wine	0.051* (0.020)	0.048* (0.022)	0.049* (0.021)	0.050* (0.021)
White wine	0.127** (0.023)	0.127** (0.025)	0.126** (0.024)	0.126** (0.024)
Organic	0.128** (0.040)	0.134** (0.042)	0.129** (0.040)	0.129** (0.040)
Alcohol by volume	0.063** (0.020)	0.067** (0.021)	0.067** (0.020)	0.067** (0.020)
ASC				
*j* = 1	1.413** (0.296)	0.844** (0.318)		
*j* = 2			-1.140** (0.304)	-1.141** (0.304)
*j* = 3			-0.968** (0.306)	-0.970** (0.306)
*j* = 4			-1.117** (0.304)	-1.118** (0.304)
*j* = 5			-1.304** (0.311)	-1.306** (0.312)
*j* = 6			-1.598** (0.311)	-1.600** (0.311)
*j* = 7			-1.802** (0.306)	-1.802** (0.306)
*j* = 8			-1.826** (0.306)	-1.828** (0.307)
*j* = 9			-1.826** (0.308)	-1.828** (0.309)
*j* = 10			-2.020** (0.308)	-2.023** (0.309)
*τ*		2.407** (0.306)		7.137** (0.000)
Log-likelihood	-9,759.748	-9,573.560	-9,533.002	-9,533.000
Observations	4,437	4,437	4,437	4,437
*K*	22	23	30	31
${\bar{\rho }}^{2}$	0.043	0.061	0.064	0.064
AIC	19,563.497	19,193.121	19,126.004	19,128.000
BIC	19,704.247	19,340.268	19,317.936	19,326.330

**Table 7:** Estimation results for treatment 4.

	RUM-OptOut	SAT-OptOut	RUM-ASCs	SAT-ASCs
Price	-0.107** (0.011)	-0.108** (0.011)	-0.106** (0.011)	-0.110** (0.011)
Country of origin				
Chile	-0.140 (0.116)	-0.143 (0.118)	-0.143 (0.116)	-0.126 (0.126)
France	-0.093 (0.086)	-0.092 (0.088)	-0.093 (0.087)	-0.067 (0.094)
Italy	0.014 (0.101)	0.023 (0.104)	0.013 (0.101)	0.093 (0.113)
New Zealand	-0.020 (0.123)	-0.026 (0.125)	-0.020 (0.123)	-0.004 (0.130)
South Africa	-0.132 (0.108)	-0.139 (0.111)	-0.131 (0.109)	-0.135 (0.117)
Spain	-0.113 (0.094)	-0.114 (0.095)	-0.115 (0.095)	-0.101 (0.099)
USA	-0.447** (0.105)	-0.452** (0.107)	-0.448** (0.106)	-0.432** (0.114)
Grape				
Cabernet Sauvignon	0.282* (0.118)	0.285* (0.119)	0.282* (0.119)	0.287* (0.124)
Chardonnay	0.484** (0.142)	0.489** (0.144)	0.480** (0.142)	0.470** (0.148)
Chenin Blanc	0.340** (0.129)	0.346** (0.131)	0.341** (0.129)	0.346* (0.138)
Malbec	0.276* (0.121)	0.282* (0.124)	0.276* (0.122)	0.306* (0.129)
Merlot	0.274* (0.114)	0.286* (0.116)	0.273* (0.115)	0.310* (0.123)
Pinot Grigio	0.430** (0.122)	0.436** (0.124)	0.427** (0.122)	0.449** (0.128)
Pinot Noir	0.350** (0.099)	0.359** (0.101)	0.352** (0.100)	0.389** (0.105)
Sauvignon Blanc	0.518** (0.150)	0.526** (0.154)	0.519** (0.151)	0.552** (0.161)
Tempranillo	0.174 (0.114)	0.179 (0.116)	0.176 (0.114)	0.194 (0.120)
Character				
Red wine	0.093** (0.027)	0.094** (0.028)	0.092** (0.027)	0.094** (0.029)
White wine	0.133** (0.036)	0.134** (0.037)	0.133** (0.036)	0.129** (0.038)
Organic	0.039 (0.059)	0.044 (0.060)	0.038 (0.059)	0.074 (0.064)
Alcohol by volume	0.036 (0.029)	0.035 (0.030)	0.036 (0.029)	0.032 (0.031)
ASC				
*j* = 1	-0.188 (0.422)	-0.254 (0.429)		
*j* = 2			0.145 (0.424)	0.240 (0.456)
*j* = 3			0.178 (0.428)	0.355 (0.459)
*j* = 4			0.258 (0.430)	0.526 (0.460)
*j* = 5			0.284 (0.434)	0.693 (0.462)
*j* = 6			0.282 (0.435)	0.856 (0.462)
*j* = 7			0.242 (0.459)	1.010* (0.487)
*j* = 8			0.147 (0.470)	1.278* (0.503)
*j* = 9			0.414 (0.453)	2.046** (0.492)
*j* = 10			0.281 (0.480)	2.892** (0.581)
*τ*		2.804** (0.557)		2.399** (0.135)
Log-likelihood	-3,670.398	-3,667.829	-3,667.282	-3,640.319
Observations	3,691	3,691	3,691	3,691
*K*	22	23	30	31
${\bar{\rho }}^{2}$	0.046	0.046	0.044	0.051
AIC	7,384.796	7,381.657	7,394.564	7,342.639
BIC	7,521.496	7,524.571	7,580.973	7,535.262

Comparing RUM-OptOut and SAT-OptOut for treatments 1–3 (standard stated preference), we see that considering satisficing leads to an improvement in fit of 3.4, 56.8 and 185.2 log-likelihood units, respectively. This suggests that even in standard data, considering satisficing under the assumption of a left-to-right search path is important. However, in the models where we estimate the full *J* − 1 set of alternative-specific constants, the story is somewhat moderated. It is clear that RUM-ASCs and SAT-ASCs across the three treatments fit the data better than both RUM-OptOut and SAT-OptOut. Furthermore, we notice that all alternative-specific constants are negative and tend to become larger (in absolute terms) as we move from left to right in the choice task. This implies that alternatives further to the right, all else equal, are less likely to be chosen relative to the ‘none-of-these-bottles’ alternative. The most notable finding when comparing RUM-OptOut and SAT-OptOut is that accommodating satisficing leads to very modest increases in model fit: 0.6, 1.4 and 2 × 10^−3^ log-likelihood units for treatments 1, 2 and 3, respectively. This is not surprising given the estimated values of *τ*, which are large relative to the other estimated parameters, indicating that no satisfactory alternative was found and a decision maker defaults to their backup strategy. As a result, support for satisficing behavior in these treatments is small. That said, we want to emphasize that in these treatments, the evaluation order is unknown and we assume that alternatives are processed in the direction of reading. We recognize that this assumption is questionable given that a decision maker might just as easily start in the middle or at the right side of the choice task. It is also increasingly likely that this assumption is violated as the number of alternatives increases (which might explain the minuscule improvement in model fit for treatment 3). This issue aside, although not directly comparable (due to potential scale differences) the marginal utility parameters estimated in both candidate models are broadly equivalent in terms of the sign, magnitude and significance.

However, our results do have important implications for the behavioral interpretation of the alternative-specific constants in both a satisficing and non-satisficing model. In the models where we estimate only the ‘none-of-these-bottles’ alternative, the large increase in model fit associated with the inclusion of *τ* suggests that it has large explanatory power. However, the fact that its inclusion when we estimate *J* − 1 alternative-specific constants does not lead to a large improvement in model fit suggests that in standard data, *τ* acts like a generative constant that explains the ordering effect normally captured by the full set alternative-specific constants when these are not included. Furthermore, in the naïve model specifications, the alternative-specific constants capture the general downward trend in choice proportions from the leftmost alternative to the rightmost alternative. But in the satisficing model, this is captured by *τ*, leaving the alternative-specific constants to capture the average influence of factors that are not explained by the attributes or the left-right processing of alternatives. Indeed, our Monte-Carlo simulations show considerable bias in the alternative-specific constants when we fail to estimate the threshold when the data generating process is satisficing.

Whereas the evaluation order was unknown in treatments 1–3, in treatment 4 it is known. When comparing RUM-OptOut and SAT-OptOut, we see that the improvement in model fit from estimating *τ* is very small. Interestingly, we see that in terms of log-likelihood value, SAT-OptOut and RUM-ASCs both comparably fit the data, but when we consider the AIC and BIC statistics SAT-OptOut fits the data much better. This further underlines the result above that *τ* is a generative constant and that its ability to explain the alternative-specific constants is much greater the more closely tied the alternative-specific constants are to the actual order in which alternatives were evaluated. Remember that in treatment 4 alternatives could only be chosen if they were in fact revealed. Thus, the fact that the alternative-specific constants are increasingly positive implies that, once they are revealed, alternatives that appear later in the sequence are more likely to be chosen. This is a logical finding given that latter alternatives are less likely to be revealed if an alternative appearing earlier in the sequence exceeded some minimum level of acceptable utility. The fact that this expectation is only corroborated in the satisficing model provides further support for its use when the evaluation order is known. Comparing RUM-ASCs and SAT-ASCs, we do see an improvement in the model fit of almost 30 log-likelihood units. While this improvement in model fit is supported even after accounting for the loss of parsimony, we admit that it is a relatively small gain compared to what could be achieved under alternative-specifications (e.g., accounting for unobserved heterogeneity). But this improvement in fit is all the more striking given the insights from the synthetic application that accounting for satisficing is unlikely to yield any substantial gains in model fit. It also reinforces the need to know the evaluation order for this type of model, only then are you likely to observe any meaningful improvement in model fit. Once more, the estimated marginal utility parameters are fairly consistent.

#### Scenario analysis

While the improvement in model fit may not be large from explicitly considering satisficing, the failure to do so has significant implications for prediction. Consider, that you manage a store and need to place bottles of wine on the shelf. Supposing the collection of nine relatively “superior” and nine relatively “inferior” bottles, as shown in Table 8. Our classification of superior” andinferior” bottles are informed by the estimated marginal utilities. Compared to the inferior bottles, the superior bottles are cheaper and have combinations of non-price attributes that were found, on average, to have higher marginal utilities. As a result, the superior bottles yield higher utility compared to the inferior bottles. The questions for you as a manager are: What bottles to place on the self? and; What is the optimal order in which to place these bottles? As an analyst, we ask a slightly longer question: What is the optimal selection *and* order in which to place the bottles conditional on how customers make decisions? The implications, as we will show, are clear. If you believe that people are utility maximizing and consider all bottles before making a choice, the order does not matter. However, if you believe that people are satisficing, then placing the bottles on the shelf as if they are utility maximizing may lead to suboptimal orderings and consequently a loss in sales revenue.

**Table 8:** Bottles considered in scenario analysis.

	Bottles A–I (superior bottles)									Bottles R–Z (inferior bottles)								
	A	B	C	D	E	F	G	H	I	R	S	T	U	V	W	X	Y	Z
Price (£)	4.00	4.00	4.00	4.50	4.50	4.50	4.50	5.00	5.50	15.50	16.00	16.50	17.00	17.00	18.00	18.00	18.00	20.00
Country of origin																		
Australia				✓	✓		✓	✓		✓		✓			✓	✓		
France	✓	✓							✓				✓	✓				
Italy			✓			✓												
USA											✓						✓	✓
Grape																		
Blend										✓		✓	✓		✓	✓		✓
Cabernet Sauvignon			✓		✓		✓		✓									
Malbec				✓				✓										
Merlot		✓				✓											✓	
Pinot Noir														✓				
Tempranillo	✓										✓							
Character (Red wine)	5	4	5	4	5	4	5	5	4	3	4	5	5	4	2	4	4	1
Organic		✓	✓	✓		✓		✓		✓					✓			
Alcohol by volume (%)	13.0	13.5	15.0	11.5	12.0	12.5	13.5	12.5	14.5	12.0	13.5	12.5	12.0	11.5	10.0	12.0	14.5	11.5

In Tables 9 and 10, we show simulation results of the optimal order based on the actual parameter estimates from each of our empirical models. Specifically, we identify the arrangement (and subset) of bottles that maximize the expected revenue, E(Revenue), generated from a single representative consumer: $E\left(\text{Revenue}\right)=\sum_{j=1}^{J}\text{Pr}\left(j|\text{X},\hat{\beta },\hat{\tau }\right)x_{pj}$, where $\text{Pr}\left(j|\text{X},\hat{\beta },\hat{\tau }\right)$ denotes the probability for alternative *j* conditional on scenarios **X** and estimated parameters $\hat{\beta }$ and, where applicable, $\hat{\tau }$; and, *x*_*pj*_ is the price of alternative *j*. For this analysis we consider bottles A–I and bottles R–Z in Table 8 and the “none-of-these-bottles” option. Results are shown in Tables 9 and 10, respectively.

**Table 9:** Predictions or expected revenue and optimal arrangement for bottles A–I (superior bottles).

	Best arrangement	Expected revenue (£) conditional on		Difference
		Utility maximization	Satisficing	
Treatment 1				
RUM-OptOut	F,H,I*	3.87 [3.70,4.04]	3.68 [3.35,3.93]	-0.19 [-0.56, 0.12]
SAT-OptOut	F,I,H	3.87 [3.70,4.04]	3.79 [3.44,4.06]	-0.09 [-0.47, 0.24]
Difference		0.00 [0.00,0.00]	0.11 [0.06,0.16]	0.11 [0.06,0.16]
RUM-ASCs	H,I,F	3.91 [3.73,4.08]	3.71 [3.30,3.94]	-0.20 [-0.64, 0.10]
SAT-ASCs	F,I,H	3.90 [3.72,4.07]	3.73 [3.33,3.96]	-0.17 [-0.60, 0.13]
Difference		-0.01 [-0.02, 0.00]	0.02 [-0.01, 0.06]	0.03 [0.00,0.07]
Treatment 2				
RUM-OptOut	D,H,I,E,F,G*	3.93 [3.78,4.06]	3.63 [3.22,3.94]	-0.29 [-0.72, 0.04]
SAT-OptOut	E,I,H,D,F,G*	3.86 [3.73,3.99]	3.83 [3.39,4.16]	-0.03 [-0.49, 0.32]
Difference		-0.06 [-0.07,-0.05]	0.20 [0.16,0.24]	0.26 [0.22,0.30]
RUM-ASCs	F,I,H,D,G,E	3.99 [3.84,4.13]	3.79 [3.56,3.95]	-0.21 [-0.47, 0.02]
SAT-ASCs	E,I,H,F,D,G	3.88 [3.74,4.01]	3.88 [3.65,4.04]	0.00 [-0.26, 0.22]
Difference		-0.11 [-0.14,-0.09]	0.09 [0.07,0.11]	0.20 [0.17,0.24]
Treatment 3				
RUM-OptOut	A,C,E,G,F,H,D,I,B*	3.87 [3.75,3.98]	4.19 [3.93,4.37]	0.31 [0.04,0.54]
SAT-OptOut	B,I,H,E,F,D,G,C,A*	3.87 [3.75,3.98]	4.19 [3.93,4.37]	0.31 [0.04,0.54]
Difference		0.00 [0.00,0.00]	0.00 [0.00,0.00]	0.00 [0.00,0.00]
RUM-ASCs	G,I,H,E,D,F,B,A,C	4.01 [3.88,4.13]	3.97 [3.85,4.09]	-0.04 [-0.20, 0.13]
SAT-ASCs	G,I,H,E,D,F,A,B,C	3.97 [3.85,4.09]	4.01 [3.88,4.13]	0.04 [-0.13, 0.21]
Difference		-0.04 [-0.06,-0.02]	0.03 [0.02,0.05]	0.07 [0.05,0.10]
Treatment 4				
RUM-OptOut	I,H,F,D,G,E,B,A,C	2.92 [1.65,4.05]	2.77 [1.59,3.87]	-0.15 [-1.81, 1.57]
SAT-OptOut	I,H,G,E,D,F,B,C,A	2.90 [1.65,3.98]	2.79 [1.59,3.87]	-0.12 [-1.80, 1.58]
Difference		-0.02 [-1.71, 1.70]	0.01 [-1.63, 1.65]	0.03 [-2.33, 2.39]
RUM-ASCs	I,H,F,G,E,D,A,C,B	2.91 [1.66,3.97]	2.73 [1.55,3.77]	-0.17 [-1.83, 1.48]
SAT-ASCs	H,I,E,D,F,G,A,C,B	2.84 [1.62,3.90]	2.77 [1.56,3.80]	-0.07 [-1.71, 1.59]
Difference		-0.07 [-1.74, 1.60]	0.03 [-1.60, 1.66]	0.10 [-2.23, 2.45]

Notes:

* signifies that there is more than one best arrangment; 95 percent confidence intervals are reported in square brackets.

**Table 10:** Predictions or expected revenue and optimal arrangement for bottles R–Z (inferior bottles).

	Best arrangement	Expected revenue (£) conditional on		Difference
		Utility maximization	Satisficing	
Treatment 1				
RUM-OptOut	U,V,Y*	8.57 [7.58,9.55]	8.50 [7.41,9.54]	-0.08 [-1.53, 1.37]
SAT-OptOut	U,Y,V	8.57 [7.58,9.55]	8.56 [7.48,9.61]	-0.01 [-1.48, 1.44]
Difference		0.00 [0.00,0.00]	0.06 [0.01,0.13]	0.06 [0.01,0.13]
RUM-ASCs	Y,V,U	8.61 [7.63,9.59]	8.39 [7.40,9.37]	-0.22 [-1.62, 1.18]
SAT-ASCs	U,V,Y	8.61 [7.63,9.58]	8.40 [7.40,9.38]	-0.21 [-1.61, 1.19]
Difference		-0.01 [-0.05, 0.03]	0.01 [-0.09, 0.11]	0.01 [-0.08, 0.12]
Treatment 2				
RUM-OptOut	T,U,V,W,Y,X*	9.99 [8.87,11.08]	10.68 [9.22,12.00]	0.68 [-1.11, 2.44]
SAT-OptOut	W,X,Y,U,V,T*	9.83 [8.75,10.87]	10.68 [9.22,12.00]	0.84 [-0.93, 2.57]
Difference		-0.16 [-0.32, 0.01]	0.00 [0.00,0.01]	0.16 [0.00,0.32]
RUM-ASCs	V,Y,U,X,T,W	9.99 [8.87,11.08]	9.98 [8.86,11.01]	0.00 [-1.55, 1.52]
SAT-ASCs	W,Y,U,V,X,T	9.83 [8.80,10.84]	10.16 [9.00,11.24]	0.33 [-1.21, 1.83]
Difference		-0.16 [-0.33, 0.02]	0.17 [0.06,0.30]	0.33 [0.12,0.54]
Treatment 3				
RUM-OptOut	R,U,X,S,T,V,Z,Y,W*	9.95 [8.78,11.10]	12.69 [11.57,13.70]	2.74 [1.17,4.28]
SAT-OptOut	Z,W,X,Y,V,U,T,S,R*	9.95 [8.78,11.10]	12.70 [11.58,13.72]	2.75 [1.18,4.29]
Difference		0.00 [0.00,0.00]	0.01 [0.01,0.02]	0.01 [0.01,0.02]
RUM-ASCs	X,V,Y,T,U,S,W,R,Z	10.04 [8.82,11.17]	9.99 [8.81,11.13]	-0.05 [-1.68, 1.65]
SAT-ASCs	X,V,Y,T,U,S,W,R,Z	9.99 [8.76,11.15]	10.05 [8.88,11.19]	0.06 [-1.56, 1.75]
Difference		-0.05 [-0.11, 0.02]	0.06 [-0.03, 0.15]	0.11 [0.00,0.22]
Treatment 4				
RUM-OptOut	V,T,X,U,Y,W,Z,R,S	7.20 [3.84,10.50]	6.86 [3.66,10.04]	-0.33 [-4.96, 4.26]
SAT-OptOut	V,T,X,U,W,Y,Z,R,S	7.05 [3.74,10.33]	6.99 [3.68,10.20]	-0.06 [-4.78, 4.54]
Difference		-0.15 [-4.70, 4.37]	0.13 [-4.27, 4.52]	0.27 [-6.17, 6.56]
RUM-ASCs	V,T,X,U,Y,W,Z,R,S	7.23 [3.71,10.68]	7.35 [3.83,10.81]	0.12 [-4.84, 5.04]
SAT-ASCs	V,T,U,Y,X,S,Z,R,W	7.10 [3.77,10.42]	7.47 [3.89,10.97]	0.36 [-4.43, 5.30]
Difference		-0.13 [-4.86, 4.48]	0.12 [-4.75, 4.92]	0.25 [-6.33, 7.10]

Notes:

* signifies that there is more than one best arrangment; 95 percent confidence intervals are reported in square brackets.

Focusing firstly on the arrangement of the superior bottles (Table 9) where self space is limited to three bottles (akin to treatment 1), there are 3!(93)=504 possible arrangements. All four models for treatment 1 identify that the optimal arrangement includes bottles F, H and I, but the optimal order differs by model. Based on the RUM-OptOut model, where utility maximizing is assumed, the optimal arrangement leads to an expected revenue of £3.87 per representative consumer. Note, however, if utility maximization is the incorrect assumption and the consumer instead adopts a satisficing decision rule, this arrangement will yield a lower expected revenue of £3.68. That is, revenue will be overpredicted by almost £0.20 per consumer when in fact the consumer satifices. If instead, the manager arranges the bottles accordingly to the SAT-OptOut model, where consumers are believed to satisfice, a revenue of £3.79 can be expected. Note that since the bottles are optimally arranged (with satisficing accounted for) this estimate is higher than the respective estimate predicted using the RUM-OptOut arrangement. This difference of £0.11 is the expected loss in revenue due to a suboptimal ordering of bottles that arises when the consumer is assumed to be a utility maximizer when in fact the consumer satisfices. It is crucial to note that there is no expected revenue loss from assuming satisficing when in fact utility maximization is adopted. That is, the optimal arrangements under RUM-OptOut and SAT-OptOut both predict the same revenue under utility maximization. This insight, leads to a straightforward recommendation for the manager to base the predictions on a satisficing model, even if utility maximizing is assumed to be the true behavior, as bottle order is less consequential for revenue predictions under utility maximization. Moreover, the mistake of wrongly assuming satisficing is small compared to the mistake of wrongly assuming utility maximizing. Turning attention to predictions arising from the RUM-ASCs and SAT-ASCs models, we observe a similar pattern. This said, the expected revenue loss of £0.02 associated with incorrectly assuming utility maximizing when in fact the true behavior is satisficing is considerably smaller. This is not surprising given that the alternative-specific constants in the RUM-ASCs model do capture the general downward trend in choosing bottles further along the shelf. Moving to the situation where self space is limited to six bottles (akin to treatment 2) the task for the manager is to identify the best arrangement out of 6!(96)=60,480 possible arrangements. Again, all four models suggest the same subset of bottles, but different orderings. The predictions retrieved from RUM-OptOut and SAT-OptOut reinforce the inferences derived from treatment 1. When alternative-specific constants are accommodated, however, the simulations suggest that the mistake of wrongly assuming utility maximizing is larger (in absolute terms) compared to the mistake of wrongly assuming satisficing. If there is space for all nine bottles (as in treatments 3 and 4) the manager’s task is identify the optimal ordering from 9! = 362, 880 possible arrangements. For these treatments, we find a somewhat different story. The differences between models are relatively small, meaning that, while the recommendation for the manager to defaultly generate predictions on the satisficing model still holds, it is of lesser consequence. The predicted difference between the two behavioral rules appears of greater relevance. For example, for the RUM-OptOut model in treatment 3, there is a difference of £0.31 per representative consumer. In short, making an incorrect assumption on the behavioral rule is more costly than using the incorrect model.

Switching attention to the optimal arrangement of inferior bottles (Table 10), we find similar results. The most notable difference, however, is that the loss of revenue from failing to consider satisficing is potentially larger in absolute terms as the number of bottles to arrange increases. However, these estimates are largely insignificant. This result is rather unsurprising. The inferior bottles of wine tend to be expensive bottles with less desirable attributes, e.g., grape variety and country of origin. Even when compared to other inferior bottles of wine, they are unlikely to be chosen under either decision rule. However, given that they are all expensive, the potential loss from an incorrect assumption about the underlying decision rule is greater in absolute terms.

Tables 9 and 10 separately identifies the optimal arrangement for superior and inferior bottles of wine, respectively. In effect, the bottles the manager has to arrange are relatively homogeneous in terms of expected utility. With this in mind, a suboptimal arrangement is likely to have a modest impact on predicted revenue. For the sake of brevity, we show the optimal arrangement when there is a mixture of superior and inferior bottles in Appendix B in S1 Appendix. As expected, the differences are generally of a much higher magnitude. Therefore, the implications of not using the correct behavioral rule are heightened when the bottles are more varied. This is an additional factor that the manager should be cognizant of.

## Conclusion

Choice modelers are increasingly interested in capturing and explaining non-utility maximizing decision processes. Several researchers have developed models to capture decision rules such as elimination-by-aspects [3] and random regret minimization [2], but few have looked at satisficing [8, 19]. In this paper, we set out to develop a simple satisficing choice model that is equally applicable to revealed and stated preference data. A satisficing individual will choose the first alternative (option) with a utility higher than some threshold level of utility. The usefulness of the model proposed in the current paper lies in its ability to explain choices. The model has the desirable property that it nests a no deliberation, or choose-the-first, strategy on the one hand and a secondary decision strategy on the other hand. The secondary decision strategy can be any the analyst deems appropriate.

We test the performance of our model using a series of Monte-Carlo simulations on data generated using the secondary decision rules and data generated using a satisficing model. We find that our satisficing model does better than the corresponding secondary decision rules at retrieving the true parameters for low to high levels of the utility threshold. For very high utility thresholds, the secondary decision rule models do just as well and are more parsimonious. That said, a pure satisficing decision rule, even one that is framed in the context of utility, is still a non-compensatory decision rule in the sense that no real trade-offs between alternatives are made. This has implications for the applicability of the model in welfare analysis for both revealed and stated preference data.

The empirical data uses a novel experimental design procedure that allows us to control the search path. Specifically, in one treatment participants received alternatives sequentially. At each point in time, respondents decided whether to choose among currently revealed alternatives or keep searching. This way of modeling satisficing is much more in line with the idea put forth by Simon [1]. An exploration of the observed choice shares revealed a large share of choices in this treatment were consistent with satisficing behavior. The model confirmed this. However, for standard treatments, where all alternatives were shown at once, the gains in model fit did not materialize. Although care is needed when drawing conclusions based on a single study, this suggests, that although our satisficing model can be applied to any revealed and stated preference data, unless the evaluation order is known its usefulness is likely to be relatively limited.

An important finding following our work is the implication for how to interpret and think about alternative-specific constants. In standard choice models that do not account for satisficing, the alternative-specific constants capture the general downward trend in choice proportions from the leftmost alternative to the rightmost alternative. But in the satisficing model, this is captured by the threshold parameter, leaving the alternative-specific constants to capture the average influence of factors that are not explained by the attributes or the left-right processing of alternatives. Furthermore, excluding all alternative-specific constants bar the one for the opt-out alternative and estimating the utility threshold suggests that the utility threshold can be viewed as a generative constant in that it captures and explains the part of the alternative-specific constants that are associated with ordering effects. Depending on the data generation process, the gain in explanatory power for estimating the threshold can be quite substantive. Furthermore, from a practical decision making standpoint, the satisficing model is better equipped to identify the optimal order of alternatives to present to a decision maker to maximize the likelihood of an alternative being chosen. For example, from a store owner’s perspective, what is the optimal order in which to place bottles on a shelf to maximize revenue? We show using simulation that a store owner assuming that their customers are satisficers can expect somewhat higher revenues compared to one that assumes they are utility maximizing.

Finally, it is acknowledged that the results are based on the condition that preferences and the threshold are homogeneous for choice observations. For obvious reasons, these are strong assumptions unlikely to hold in reality. To fully capture satisficing behavior, easy extensions to the model involve accounting for preference heterogeneity and the reparameterization of the threshold to accommodate search costs; observed and unobserved individual-specific factors that may affect the likelihood of satisficing; or aspiration levels to allow for more explicit updating of the threshold utility in response to learning. Although this is expected that this facilitates the estimation of the utility thresholds and, in doing so, better explains the presence of satisficing behavior, more research to properly investigate these aspects is warranted.

## Supporting information

S1 Appendix (PDF)
