# Supplementary material for: A simple satisficing model
Source: PLoS One. 2022 Oct 10;17(10):e0275339. doi: 10.1371/journal.pone.0275339 (PMC9550030; doi:10.1371/journal.pone.0275339)
Supplement: S1 Appendix — (PDF) [file pone.0275339.s001.pdf]

Appendix A Observed choice shares by decision rule in synthetic applications

In Fig A.1, we compare the average share (across the 1,000 sample simulations) of simulated choices that are consistent with the secondary decision strategies as a function of the level of the threshold. Each panel in Fig A.1 corresponds to a decision rule. This helps determine how well the simulated choices reflect the actual behavioral rule used when generating the data and, more importantly, to make comparisons across the different simulation treatments.

Fig A.1. Share of choices consistent with secondary decision rule

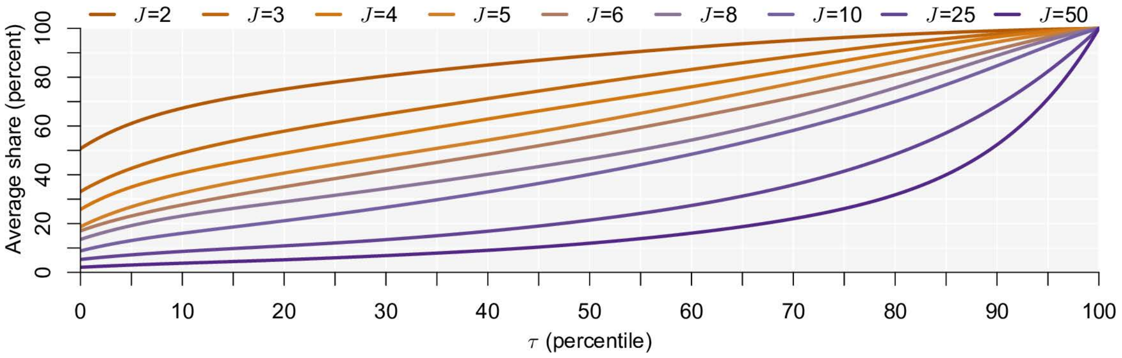

(a) Data generated on basis of choose the utility maximizing alternative secondary decision rule

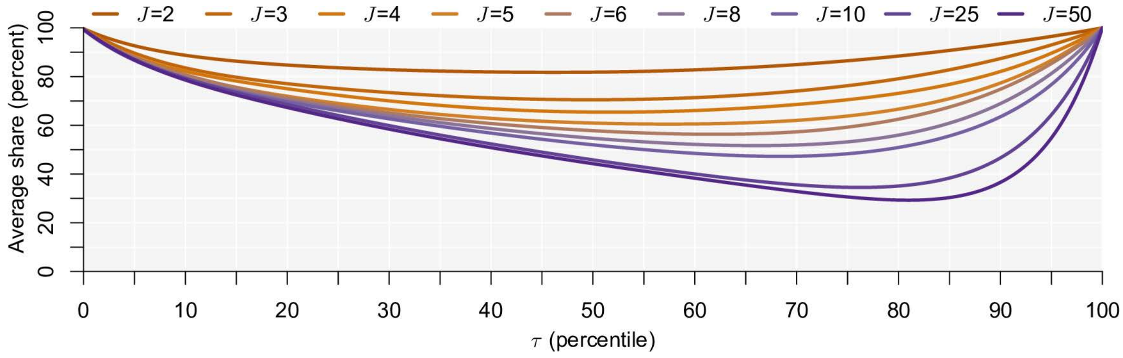

(b) Data generated on basis of choose the opt-out alternative secondary decision rule

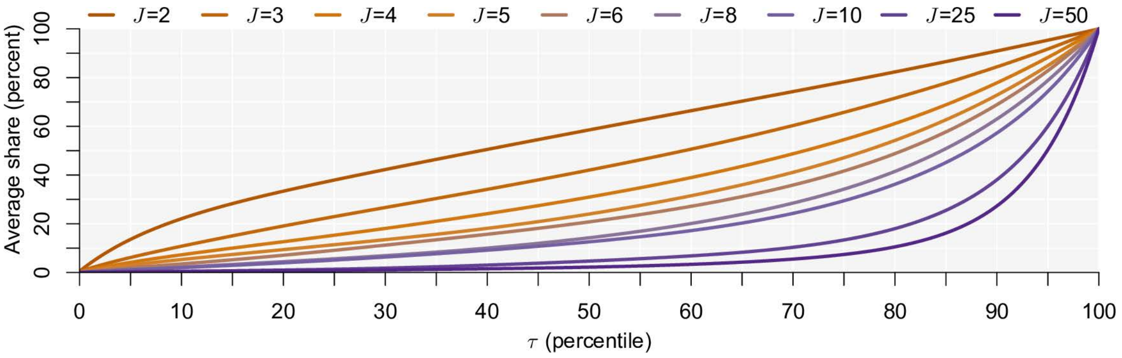

(c) Data generated on basis of choose the last alternative secondary decision rule

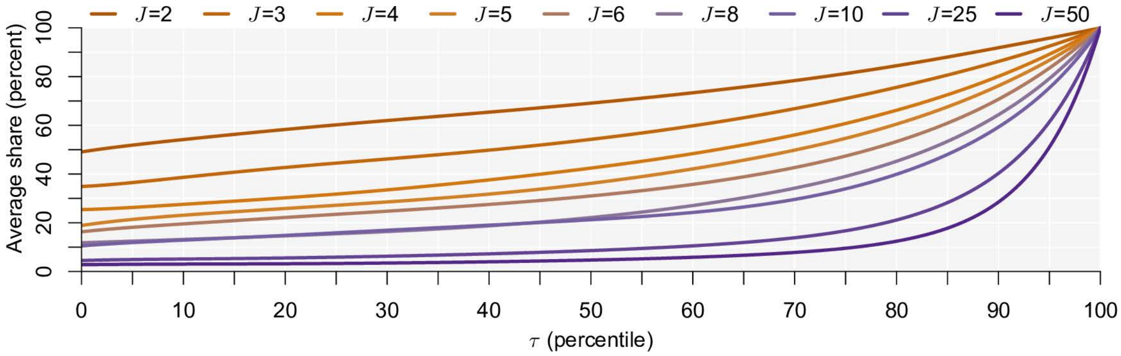

(d) Data generated on basis of choose a random alternative secondary decision rule

At sufficiently low thresholds, under both choosing the utility maximizing (Fig A.1a) and random alternative (Fig A.1d), we observe that approximately $1/J$ could be due to the secondary decision rule with the remaining, i.e., $(J-1)/J$, being explained by satisficing. However, as presented in the paper, as the threshold increases the share of choices that are consistent with satisficing decreases, leading to an increase in the share of choices that match these secondary decision rules. For the choose the last backup strategy (Fig A.1c), we find a similar result. However, with very low thresholds the share of choices that are consistent with this backup strategy is essentially zero since the chances of encountering an alternative with a utility greater than or equal to the threshold utility before reaching the final alternative is high. But this probability reduces as the threshold increases, leading to a higher share of decision makers choosing the final alternative. Looking at the share of choices that are consistent with the opt-out alternative (Fig A.1b), we see a u-shaped pattern. Remember, we are only looking at the case where the opt-out is the first alternative encountered; the case where the opt-out is encountered last is captured by the "choose the last" strategy. The logic behind this u-shape is as follows. When the threshold is sufficiently low, this strategy is indistinguishable from satisficing, i.e., both involve choosing the first alternative encountered. As the threshold increases, the likelihood that the first alternative exceeds the threshold reduces. However, as the threshold increases the likelihood that any of the alternatives exceeds the threshold falls meaning that decision makers will use their secondary decision strategy, which in this case is to choose the opt-out (i.e., the first) alternative. Across all decision rules, for a given threshold the share of choices consistent with the backup strategy reduces as the number of alternatives increase. This is driven by the obvious higher chance of encountering an alternative that yields utility greater than the threshold as the number of alternatives increase.

Appendix B Additional scenario analysis

In Table B.1, we show additional scenario analysis when we allow for a mix of superior and inferior wine bottles. The interpretation of the table remains as presented in the paper. As is clear from the table, ordering becomes more important whenever we have a mix of bottles. This is because the difference in price is more pronounced and a more optimal order can induce respondents to choose a slightly more expensive bottle leading to higher sales revenues for the store owner, and potentially higher profits.

Table B.1. Optimal arrangement of superior and inferior bottles (Bottles A–E and W–Z)

		Expected revenue (£) conditional on		
	Best arrangement	Utility maximization	Satisficing	Difference
Treatment 1				
RUM-OptOut	X,W,Y*	8.12 [7.12,9.15]	8.05 [7.00,9.09]	-0.07 [-1.57, 1.37]
SAT-OptOut	W,Y,X	8.12 [7.12,9.15]	8.10 [7.02,9.17]	-0.02 [-1.53, 1.46]
Difference		0.00 [0.00,0.00]	0.05 [-0.04, 0.17]	0.05 [-0.04, 0.17]
RUM-ASCs	X,Y,W	8.13 [7.12,9.16]	7.92 [6.91,8.95]	-0.21 [-1.64, 1.26]
SAT-ASCs	W,Y,X	8.12 [7.11,9.15]	7.93 [6.93,8.96]	-0.19 [-1.60, 1.28]
Difference		-0.01 [-0.03, 0.01]	0.02 [-0.02, 0.09]	0.02 [-0.02, 0.10]
Treatment 2				
RUM-OptOut	D,E,W,Y,X,Z*	6.27 [5.64,6.93]	5.16 [4.52,5.78]	-1.11 [-2.02,-0.23]
SAT-OptOut	E,Z,Y,X,W,D	5.19 [4.78,5.65]	6.77 [5.79,7.75]	1.58 [0.52,2.65]
Difference		-1.08 [-1.33,-0.85]	1.61 [1.24,2.03]	2.69 [2.24,3.18]
RUM-ASCs	W,Y,X,Z,E,D	6.94 [6.19,7.76]	5.21 [4.75,5.71]	-1.73 [-2.67,-0.82]
SAT-ASCs	E,X,Y,W,Z,D	5.36 [4.89,5.90]	6.90 [6.08,7.77]	1.54 [0.55,2.52]
Difference		-1.58 [-1.91,-1.26]	1.70 [1.27,2.17]	3.27 [2.74,3.85]
Treatment 3				
RUM-OptOut	A,B,C,D,Y,E,W,Z,X*	4.74 [4.41,5.12]	5.92 [5.39,6.51]	1.17 [0.52,1.85]
SAT-OptOut	B,Z,W,X,Y,E,D,C,A*	4.74 [4.41,5.12]	5.92 [5.39,6.51]	1.17 [0.52,1.85]
Difference		0.00 [0.00,0.00]	0.00 [0.00,0.00]	0.00 [0.00,0.00]
RUM-ASCs	W,Y,X,Z,D,E,A,B,C	5.53 [4.98,6.16]	5.40 [4.89,5.98]	-0.13 [-0.95, 0.66]
SAT-ASCs	W,Y,X,Z,D,E,A,B,C	5.28 [4.78,5.85]	5.53 [4.98,6.17]	0.26 [-0.54, 1.06]
Difference		-0.25 [-0.37,-0.15]	0.14 [0.06,0.23]	0.39 [0.26,0.54]
Treatment 4				
RUM-OptOut	X,Y,W,Z,D,E,A,B,C	4.85 [2.52,7.25]	4.64 [2.43,6.92]	-0.21 [-3.52, 3.11]
SAT-OptOut	X,Y,W,Z,D,E,A,B,C	4.81 [2.51,7.24]	4.68 [2.47,7.01]	-0.13 [-3.37, 3.11]
Difference		-0.04 [-3.18, 3.05]	0.03 [-3.01, 3.08]	0.07 [-4.25, 4.44]
RUM-ASCs	X,Y,W,Z,D,E,A,B,C	4.81 [2.57,7.21]	3.67 [2.00,5.34]	-1.14 [-4.04, 1.61]
SAT-ASCs	X,Y,W,Z,D,E,C,A,B	4.02 [2.17,5.84]	4.67 [2.46,7.13]	0.65 [-2.22, 3.72]
Difference		-0.79 [-3.63, 2.00]	1.00 [-1.65, 3.84]	1.79 [-2.05, 5.77]

Notes: * signifies that there is more than one best arrangment; 95 percent confidence intervals are reported in square brackets.
